# A method for statistical analysis of repeated residential movements to link human mobility and HIV acquisition

**DOI:** 10.1371/journal.pone.0217284

**Published:** 2019-06-05

**Authors:** Adrian Dobra, Till Bärnighausen, Alain Vandormael, Frank Tanser

**Affiliations:** 1 Department of Statistics, University of Washington, Seattle, WA, United States of America; 2 Heidelberg Institute of Global Health (HIGH), Medical Faculty and University Hospital, Heidelberg University, Heidelberg, Germany; 3 Department of Global Health and Population, Harvard T.H. Chan School of Public Health, Boston, United States of America; 4 Africa Health Research Institute,KwaZulu-Natal, South Africa; 5 School of Nursing and Public Health, University of KwaZulu-Natal, Durban, South Africa; 6 Centre for the AIDS Programme of Research in South Africa (CAPRISA), University of KwaZulu-Natal, Durban, South Africa; 7 Research Department of Infection & Population Health, University College London, London, United Kingdom; Johns Hopkins Bloomberg School of Public Health, UNITED STATES

## Abstract

We propose a method for analyzing repeated residential movements based on graphical loglinear models. This method allows an explicit representation of residential presence and absence patterns from several areas without defining mobility measures. We make use of our method to analyze data from one of the most comprehensive demographic surveillance sites in Africa that is characterized by high adult HIV prevalence, high levels of poverty and unemployment and frequent residential changes. Between 2004 and 2016, residential changes were recorded for 8,857 men over 35,500.01 person-years, and for 12,158 women over 57,945.35 person-years. These individuals were HIV negative at baseline. Over the study duration, there were a total of 806 HIV seroconversions in men, and 2,458 HIV seroconversions in women. Our method indicates that establishing a residence outside the rural study area is a strong predictor of HIV seroconversion in men (OR = 2.003, 95% CI = [1.718,2.332]), but not in women. Residing inside the rural study area in a single or in multiple locations is a less significant risk factor for HIV acquisition in both men and women compared to moving outside the rural study area.

## Introduction

This paper is concerned with modeling repeated residential movements of a group of individuals over a certain period of time, and with the assessment of the predictive associations between these multivariate patterns of residential changes and health outcomes of interest such as HIV acquisition. To a good extent, the statistical literature on human mobility has focused on the estimation of migration flows [1–3]. Migration flows are represented as origin-destination migration flow tables. These are square tables in which the rows and columns correspond with areas of interest. The (*i*, *j*) cell contains a count of the number of individuals that left from area *A*_*i*_ and moved to area *A*_*j*_ over the course of a specified time frame. The inclusion of other categorical variables lead to higher-dimensional migration flow tables. However, migration flow tables cannot capture the movement of those individuals that resided in more than three areas during the time frame of observation. An example individual that left from an area *A*_1_ to move to another area *A*_2_, then moved again to area *A*_3_, would contribute with a count of 1 to the (1, 2) and (2, 3) cells of the resulting migration flow table. But, the link between residential movements that are associated with the same individual will be lost.

Other important classes of statistical models for human mobility are Lèvy flights models [4] and multiplicative latent factor models [5]. Lèvy flights models make use of a power law to represent the probability that an individual changes their residence over a certain distance. Under this model, moving over a shorter distance is more likely than moving over longer distances, but residential movements over longer distances can still take place even if they occur less often. Multiplicative latent factor models improve the Lèvy flights models with their ability to quantify the desirability of residing in certain areas over other areas. Both the Lèvy flights models and the multiplicative latent factor models are based on the crude assumption that human travel can be seen as a Markov process in which the probability of residing in an area depends only on area in which the previous residence was located, and does not depend on the locations of previous residences. However, it is possible that individuals move repeatedly across multiple areas over longer time periods of several years. Markov process models break residential trajectories that involve multiple residential locations into pairs of consecutive locations of residency, and, by doing so, loose key dependencies that are induced by multiple locations of residency of the same individuals in a reference time frame.

Information about residential locations has also been used in statistical models through the construction of mobility measures—see, for example, [6] and the references therein. These measures are summaries of distances between consecutive residencies, or of time spent in certain locations. While mobility measures can be successfully used as independent variables in a wide range of statistical models, the connection between these measures and the areas in which individuals have resided is lost. The method for analyzing repeated residential movements we follow in this paper allows an explicit representation of residential presence and absence patterns from several areas without defining mobility measures. As such, this method offers a new perspective on what can be learned from this important type of human mobility data.

We assume that the residential locations of *N* individuals belong to *K* areas denoted by {*A*_1_, *A*_2_, …, *A*_*K*_}. For each individual, we know which areas they resided in. These data can be represented as a *N* × *K* mobility matrix *M* = (*m*_*nk*_), where

$$\begin{array}{c} m_{nk}=\{\begin{array}{cc} 1, & \;\text{if}\;\text{individual}\;n\;\text{resided}\;\text{in}\;\text{area}\;k,\\ 0, & \;\text{if}\;\text{individual}\;n\;\text{did}\;\text{not}\;\text{reside}\;\text{in}\;\text{area}\;k. \end{array} \end{array}$$

Our framework does not impose any constraints on the number of individuals *N*, or on the number of areas *K*. Other categorical variables of interest can be recorded as additional columns in the mobility matrix *M*.

By counting the number of times the same combination of levels of the categorical variables in *M* appear as rows of this matrix, a multi-dimensional contingency table is formed [7]. We propose representing the multivariate patterns of associations in this contingency table with graphical loglinear models that are a special type of hierarchical loglinear models [8, 9]. These models are determined by graphs that have vertices associated with each area. They characterize the multivariate dependency structure (e.g., independence or conditional independence) among random variables using graphs [9]. The complete subgraphs of these graphs define interaction terms of joint presence and absence patterns from two, three or several areas. A missing edge between two areas means that, conditional on presence or absence in the rest of the areas, the presence or absence of a random individual in the first area is independent of the presence or absence of the same individual in the second area.

A key step in data analysis with graphical loglinear models consists of the estimation of the underlying graph. This is called the structural learning problem [10, 11], and it becomes a very difficult computational problem when many random variables are involved [12, 13]. Bayesian methods provide a flexible framework for incorporating uncertainty of the graph structure: inference and estimation are based on averages of the posterior distributions of quantities of interest, weighted by the corresponding posterior probabilities of graphs [14]. Here we follow a Bayesian approach for solving the structural learning problem.

The goal of our statistical analysis is to identify graphs that have vertices associated with each area in the corresponding graphical loglinear models. Based on this approach, we examine the predictive value of residential locations as a driver of HIV transmission risk in a comprehensive population-based demographic surveillance site in the KwaZulu-Natal Province, South Africa—the Africa Centre, now Africa Health Research Institute (AHRI) [15]. Specifically, we analyze mobility patterns of 21,015 individuals who were HIV negative at baseline, and were registered in the AHRI demographic surveillance system. Their mobility patterns are defined by residential histories over the study period. The AHRI site is characterized by high adult HIV prevalence (24% in adults aged 15 years 30 and older in 2011), and high levels of poverty and unemployment (in 2010, 67% of adults over the age of 18 in the rural study area were unemployed) [16]. The geographical location of this demographic surveillance area is ideal for our aim.

## Background

Historically, human mobility has been one of the key drivers in the spread of HIV at a global scale [17–29]. Many studies have provided significant evidence linking increased population mobility with multiple sexual partners, reduced condom use, increased risky behavior (e.g., encounters with commercial sex workers, engaging in transactional sex) [30–32], increased sexual behavior [20, 33–38], and increased likelihood of HIV acquisition [6, 28, 39]. Mining settlements, transport corridors, or poor urban or periurban communities exacerbate the effect of the risk factors of HIV acquisition [28, 40, 41]. It has been empirically demonstrated that an individual’s risk of acquisition of HIV is strongly driven by community-level HIV prevalence [16], community-level migration intensity [42], mean number of sexual partners in the surrounding local community [43], as well as ART coverage and population viral load in the local community, respectively [16, 44]. These community-level risk factors confer substantial additional risk of new HIV infection after controlling for a suite of well-established individual-level risk factors.

In South Africa, which is the focus of this study, the risk of HIV infection has been shown to be increased by human mobility [19, 45, 46]. South Africa is one of the countries with the highest burden of HIV, and has a long history of internal labor migration of men that periodically leave their areas of permanent residence to seek temporary employment in mines and factories due to the scarcity of local employment [47]. During the apartheid era which imposed travel restrictions for Blacks, women were typically left behind to take care of families, while men submitted remittances back to their households. Because of economic conditions, this way of life continues to exist in poor rural regions of South Africa including this rural study community. However, as opposed to the aparheid era, in the last decade both men and women frequently establish residencies for various periods of time to work or for many other reasons in locations within the KwaZulu-Natal Province (e.g., Richards Bay or Durban), or in other more distant locations in South Africa (e.g., Johannesburg, Pretoria or Cape Town) [6].

The rapid increase in the adult HIV prevalence in South Africa, from 0.7% in 1990 to 13% in 2000 [48, 49], is broadly consistent with ongoing patterns of circular labor migration within the country and increased in-migration from neighboring countries after the collapse of Apartheid [50–52]. For example, a phylogenetic study from the KwaZulu-Natal province reveals that external introductions in the early 1990s, via human movement from neighboring countries, played a vital role in driving the early HIV epidemic [53]. Historically, patterns of circular migration in South Africa were shaped by the migrant labor policies of the Apartheid system. From the 1950s until the democratic transition in 1994, Apartheid authorities sought to consolidate white rule by developing urban centers and resettling black Africans into rural and undeveloped homelands. Racial segregation and resettlement was seen as a more rational distribution of African labor between white cities, industries, mines, and farms [54]. Men had to migrate from their homeland residencies to their work place for long periods of time, without the possibility of their families joining them [55]. Because of separate spheres of living, migrant men took other partners and formed second families at the places where they worked [56, 57], thus increasing the risk of HIV infection and the probability of transmission upon returning home. Apartheid policies had a profound effect on the stability of the family system, a demographic reality that drove the spread of HIV in the 1990s and thereafter.

Efforts to contain the HIV epidemic after 2000 were stalled by the South African government’s refusal to make ART available at public health-care facilities nationwide [58, 59]. This refusal was motivated by AIDS denialism among government officials, who claimed that HIV was not the cause of AIDS, that ART was toxic, and that the spread of HIV was being over-sensationalized [60, 61]. During this time, the adult HIV prevalence increased to 15.2% [49] and was as high as 29.5% among pregnant women attending antenatal clinics [48]. Following public pressure from AIDS activists and civil society organizations, the South African government made ART with a CD4+ T-cell count eligibility criteria of <200 cells/*μ*L in 2004 [62]. In 2010, treatment eligibility was extended to pregnant woman with CD4+ T-cell counts <350 cells/*μ*L and patients with active tuberculosis [62]. By 2012, the HIV prevalence among 15–49 year-olds was at 18.8% [63] and at 20.6% in 2017 [64].

## Methods

### Study setting

The study was conducted in the Africa Health Research Institute (AHRI) Population Intervention Platform Study Area (PIPSA), formerly the Africa Centre Demographic Information System (ACDIS), in uMkhanyakude District, KwaZulu-Natal Province. PIPSA was commissioned in 2000 by the Wellcome Trust as a platform for longitudinal population based studies of epidemiology and intervention research. This rural study area covers 438 km^2^, and comprises approximately 11,000 households with 100,000 individuals. This community is characterized by high HIV prevalence frequent migration, low marital rates, late marriage especially for men, polygamous marriages and multiple sexual partnerships, as well as by poor knowledge and disclosure of HIV status [15, 39, 56, 65]. Incidence peaked at 6.6 per 100 person-years in women aged 24 years, and at 4.1 per 100 person-years in men aged 29 years over the same period [39].

For over 15 years, PIPSA has continuously collected longitudinal surveillance data on a range of health care and social intervention exposures, as well as health, socio-economic and behavioral outcomes [15]. During the household data collection cycle, households are visited every 6 months by fieldworkers and information supplied by a single key informant. Population-based HIV surveillance and sexual behavior surveys take place annually. Since 2003, annual HIV testing became part of household surveillance.

### Study eligibility

Starting with 2007, all adults and adolescents aged 15-17 residing in the rural study area who were able to provide written consent were eligible to participate in the study. From 2003 to 2006, eligibility was restricted to women aged 15-49 years and men aged 15-54. We note that, although individuals under 18 are legal minors, under South African law, they can consent independently to medical treatment from the age of 14. Minors can legally consent independently to an HIV test from the age of 12, when it is in their best interest, and below the age of 12 if they can understand the benefits, risks and social implications of an HIV test [66].

### Ethics statement

Informed written consent was obtained from all eligible individuals. After signing the consent, eligible participants are interviewed in private by trained fieldworkers, who also extract blood from consenting individuals by finger-prick for HIV testing, prepare dried blood spots for HIV testing according to the Joint United Nations Programme on HIV/AIDS (UNAIDS) and World Health Organization (WHO) Guidelines for Using HIV Testing Technologies in Surveillance [15]. Ethics approval for data collection and use was obtained from the Biomedical Research and Ethics Committee (BREC) of the University of KwaZulu-Natal (Durban, South Africa), BREC approval number BE290/16. The BREC was aware that some of the study participants were legal minors, and approved the age range of participation and the specific consent procedure for minors.

### Cohort description

From the entire population under surveillance in PIPSA between January 1, 2004 and December 31, 2016, we selected those individuals who consented to test at least twice for HIV after the age of 15, and whose first test was negative. Although the annual participation rates in HIV testing are not high (see Table 1), a number of 8,857 men and 12,158 women satisfy these inclusion criteria. Participants seldom test every year, and, in this cohort, the median time between the last HIV negative and the first HIV positive tests in men was 3.34 years (IQR = 4.64), and in women 2.58 years (IQR = 3.69). The date of HIV seroconversion was assumed to occur according to a uniformly random distribution between the date of the last negative and first positive HIV test [67]. Here seroconversion refers to the transition from infection with the HIV virus to the detectable presence of HIV antibodies in the blood. Fig A from S1 Supporting Information gives the crude annual consent rates, while Fig B from S1 Supporting Information shows the consent rates by age group and gender. Although the overall consent rate changes over time, there does not seem to be any relevant difference in consent by sex and age.

**Table 1:** Annual summaries of eligibility and consent for the study participants.

Year	Eligible and Present^†^		Present and Tested^‡^		Ever Tested^¶^
	no./total no.	%	no./total no.	%	%
2005	24,317/29,690	81.9	21,359/24,317	87.8	37.6
2006	22,505/29,314	76.8	20,676/22,505	91.9	49.5
2007	21,372/29,510	72.4	19,080/21,372	89.3	53.9
2008	23,107/31,846	72.6	21,251/23,107	92.0	55.1
2009	20,197/28,957	69.7	18,211/20,197	90.2	59.3
2010	24,797/32,447	76.4	19,828/24,797	80.0	58.4
2011	24,677/29,915	82.5	18,991/24,677	77.0	61.4
2012	22,872/29,245	78.2	16,939/22,872	74.1	59.1
2013	22,438/28,642	78.3	18,360/22,438	81.8	63.9
2014	21,443/28,194	76.1	17,850/21,443	83.2	65.8
2015	21,441/27,587	77.7	19,866/21,441	92.7	72.8
2016	14,757/18,075	81.6	13,438/14,757	91.1	75.6

^†^ Shows the number of study participants eligible for testing (denominator) and the number present on the date of the household visit (numerator).

^‡^ Shows the number of study participants present at the household visit (denominator) and the number that consented to an HIV test (numerator).

^¶^ Shows the percentage of study participants that had at least one HIV test over the observation period. Summaries have not been produced for the first year of the study (2004).

PIPSA collects data about all the individuals that are members of a family unit or a household in the rural study area irrespective of the current residency status. It collects longitudinal residential information about the exact periods of time each study participant spent living in each location. Fieldworkers record changes in residency as the origin place of residence, the destination place of residence and the date of the move. Residencies can be located inside or outside the rural study area. The residential locations inside the rural study area have been comprehensively geolocated to an accuracy of <2m [68]. Repeat-testers can change their place of residence multiple times: they can move between two residencies located inside the rural study area, between two residencies located outside the rural study area, or between a residency inside the rural study area and another residency outside the rural study area. The relevance of looking whether repeat-testers have resided outside the rural study area comes from the findings of Dobra et al. [6]. Their results indicate that, for the same rural study area, the risk of HIV acquisition is significantly increased for both men and women when they spend more time outside the rural study area, or when they change their residencies over longer distances.

For the purpose of this study, the geolocations of the homesteads have been mapped into 45 non-overlapping communities that cover the rural study area—see Figs E and F in S1 Supporting Information. The division of the rural study area into communities is motivated by the results of Tanser et al. [69]. Their study identified a significant geographical variation in HIV incidence in the same rural study area. Specifically, they identified three large irregularly-shaped clusters of new HIV infections. Although these clusters cover only 6.8% of the rural study area, about 25% of the sero-conversions that occurred over this study’s period are associated with residencies in them. This suggests the existence of clear corridors of HIV transmission inside the rural study area. Together, the results of Dobra et al [6] and Tanser et al [69] indicate that men and women who reside outside the rural study area, or occupy residencies that are located in the corridors of HIV transmission inside the rural study area are at an increased risk of acquiring HIV.

We note that the exposure period for a repeat-tester starts at the time of their first HIV test, and ends at their HIV seroconversion date for seroconverters, or at the time of their last HIV negative test for those that did not seroconvert. The residential locations occupied before seroconversion coud have contributed to changes in sexual behavior that led to HIV acquisition, while residential locations occupied after seroconversion could be associated with repeat-testers seeking family support, health care or moving away to avoid social stigma [22, 38]. For this reason, the residential locations occupied by seroconverters after they acquired HIV were discarded.

### Statistical analyses

We determined in which of the 45 communities each of the 8,857 men and 12,158 women lived in during the study period. This information was recorded as binary variables C1, C2, …, C45 with levels “yes” or “no” in two mobility matrices, one for men and one for women. We also determined whether a repeat-tester moved outside the rural study area. This information was recorded as a binary variable *Outside* with levels “yes” or “no”. Furthermore, we determined whether a repeat-tester has seroconverted, and whether a repeat-tester was younger than 30 years at start of their observation period. This information was recorded as two additional binary variables *Seroconverted* and *Young* with levels “yes” or “no”. For example, a repeat-tester that lived in communities C1 and C2, moved outside the rural study area, was older than 30 years at baseline, and has seroconverted, would have C1 = C2 = *Outside* = *Seroconverted* = yes and C3 = … = C45 = *Young* = no.

The data in the resulting mobility matrices involve 48 binary variables. The mobility matrix for men is available in S1 Data, and the mobility matrix for women is available in S2 Data. They define two dichotomous contingency tables with 2^48^ cells, one table for men and another table for women. These tables which we call mobility tables are hyper-sparse: most of their counts are zero. The mobility table for men has only 598 positive counts—see Table E in S1 Supporting Information. Among these counts, there are 292 (48.83%) counts of 1, 48 (8.03%) counts of 2, 30 (5.02%) counts of 3, 28 (4.68%) counts of 4, and 13 (2.17%) counts of 5. The top five largest counts are 192, 186, 180, 177 and 168, respectively. They correspond with men that were less than 30 years old at the start of their observation period, did not seroconvert by the end of their observation period, never moved outside the rural study area, and lived in exactly one of these communities: C7, C37, C40, C39 and C22. The mobility table for women has only 939 positive counts—see Table F in S1 Supporting Information. Among these counts, there are 534 (56.87%) counts of 1, 98 (10.44%) counts of 2, 30 (3.19%) counts of 3, 15 (1.60%) counts of 4 and 15 (1.60%) counts of 5. The top five largest counts are 185, 176, 175, 172 and 171. They correspond with women that were less than 30 years old at the start of their observation period, did not seroconvert by the end of their observation period, never moved outside the rural study area, and lived in exactly one of these communities: C22, C10, C25, C39, and C7, respectively. Tables A and B in S1 Supporting Information give the cross-classification of the men and women in the study with respect to the binary variables *Seroconverted*, *Young* and *Outside*. These tables have 2^3^ = 8 cells, and are the three dimensional marginal tables of the 48 dimensional mobility tables.

### Statistical modeling framework

In this paper we make use of a Bayesian framework for solving the structural learning problem that is suitable for the analysis of hyper-sparse contingency tables with *p* = 48 variables. This framework [70] determines graphical loglinear models that are a special type of hierarchical loglinear models [8, 9]. A graphical model for a random vector **X** = (*X*_1_, *X*_2_, …, *X*_*p*_) is specified by an undirected graph *G* = (*V*, *E*) where *V* = {1, …, *p*} are vertices or nodes, and *E* ⊂ *V* × *V* are edges or links [9]. A vertex *i* ∈ *V* of *G* corresponds with variable *X*_*i*_. The absence of an edge between vertices *i* and *j* in *G* means that *X*_*i*_ and *X*_*j*_ are conditional independent given the remaining variables *X*_*V*\{*i*,*j*}_. The graph *G* also has a predictive interpretation. Denote by nbd_*G*_(*i*) = {*j* ∈ *V*: (*i*, *j*) ∈ *E*} the neighbors of vertex *i* in *G*. Then *X*_*i*_ is conditionally independent of $X_{V\backslash ({\text{nbd}}_{G}\left(i\right)\cup \left\{i\right\})}$ given $X_{{\text{nbd}}_{G}\left(i\right)}$ which implies that, given *G*, a mean squared optimal prediction of *X*_*i*_ can be made from the neighboring variables $X_{{\text{nbd}}_{G}\left(i\right)}$. The structural learning problem estimates the structure of *G* (i.e., which edges are present or absent in *E*) from the available data **x** = (*x*^(1)^, …, *x*^(*n*)^) by sampling from the posterior distribution of *G* conditional on the data **x**, i.e.

$$\begin{array}{c} \text{Pr}\left(G|\text{x}\right)=\frac{\text{Pr}(G)\text{Pr}(\text{x}|G)}{\sum_{G\in G_{p}}\text{Pr}\left(G\right)\text{Pr}\left(\text{x}|G\right)}, \end{array}$$ (1)

where Pr(*G*) is a prior distribution on the graph space $G_{p}$ with *p* variables, and Pr(**x** | *G*) is the marginal likelihood of the data conditional on *G* [10]. We use a prior on the space of graphs $G_{p}$ that encourages sparsity by penalizing for the inclusion of additional edges in the graph *G* = (*V*, *E*) [10]:

$$\begin{array}{c} \text{Pr}\left(G\right)\propto (\frac{\beta }{1-\beta }{)}^{|E|}=(\prod_{i=1}^{p}{\left(\frac{\beta }{1-\beta }\right)}^{|{\text{nbd}}_{G}\left(i\right)|}{)}^{1/2}, \end{array}$$ (2)

where *β* ∈ (0, 1) is set to a small value, e.g. $\beta =1/(\begin{array}{c} 48\\ 2 \end{array})\approx 0.00089$. Under this prior, the expected number of edges for a graph is 1. This means that sparser graphs with few edges receive larger prior probabilities compared with denser graphs in which most edges are present.

Determining the graphs with the highest posterior probabilities (1) is a complex problem since the number of possible undirected graphs $2^{\left(\begin{array}{c} p\\ 2 \end{array}\right)}$ becomes large very fast as *p* increases. For example, our two mobility tables involve *p* = 48 variables, and the number of possible undirected graphs in $G_{48}$ is approximately 10^325^. This motivated the development of computationally efficient search algorithms for exploring large spaces of graphs that have the ability to move quickly towards high posterior probability regions by taking advantage of local computation. Among them, the birth-death Markov chain Monte Carlo (BDMCMC) algorithm [70] determines graphical loglinear models. BDMCMC is a trans-dimensional MCMC algorithm that is based on a continuous time birth-death Markov process [71]. Its underlying sampling scheme traverses $G_{p}$ by adding and removing edges corresponding to the birth and death events. This algorithm is implemented in the package BDgraph [72, 73] for R [74].

By employing the BDgraph package, we ran the BDMCMC algorithm for 250,000 iterations to sample graphs from the posterior distribution (1) on $G_{48}$ for the mobility tables for men and women. Figs C and D in S1 Supporting Information give the estimated posterior inclusion probabilities of the $(\begin{array}{c} 48\\ 2 \end{array})=1128$ edges across iterations. We see that, after about 50,000 iterations, the subsequent posterior edge inclusion estimates stabilize. For this reason, the first 50,000 sampled graphs were discarded as burn-in, and the remaining 200,000 sampled graphs were used to estimate posterior edge inclusion probabilities.

### Limitations

Representing residential locations data as mobility matrices leads to information loss, as follows: (i) the order in which an individual resides in two or more areas is no longer accounted for; (ii) residential movements that occur within the same area are missed; (iii) the amount of time an individual maintains a residence in the same area is overlooked; and (iv) the number of times an individual establishes a residence in the same area is lost. Although this loss of information can be seen as significant, the major advantage of our proposed methodology for analyzing repeated residential movements is its ability to capture repeated presence and absence patterns from several areas. For this purpose, mobility matrices suffice.

Another limitation is related to the graphs identified by structural learning in graphical loglinear models. The prior on the graph space (2) gives the same probability of existence of an edge between any two areas irrespective of the actual spatial distance between them. In this application, the use of this prior is justified: there is no reason to assume that more distant areas are less likely to be connected than areas that are closer to each other. In fact, as we will see in the Results section, the repeat-testers were more likely to make residential movements between more distant locations (e.g., a location inside the rural study area and another location outside the rural study area) than between less distant locations (e.g., two locations inside the rural study area). As such, while specifying a prior on the graph space that takes actual physical distances between areas into account is mathematically possible [75], the use of this type of spatial prior in this study was not necessary.

A third limitation of our study is related to mapping the locations inside the rural study area into 45 communities (spatial units), and of all the locations outside the rural study area into an additional spatial unit. These specific choices could induce biases related to the modifiable areal unit problem (MAUP) [76, 77]. MAUP identifies the inevitable statistical bias that occurs due to scale (i.e., different sized spatial units) and zoning (i.e., different definitions of boundaries used to define spatial units). Due to MAUP, altering the choices of spatial units employed in a statistical analysis could potentially affect the results reported in a significant manner. However, in our application, the spatial units employed were not arbitrary: the 45 communities have not been defined for the purpose of this study alone. Instead, these communities were employed in several studies conducted in AHRI/PIPSA—see, for example, [69]. These communities have specific social, economic and demographic relevance for the rural study area. For this reason, reporting results based on spatial units constructed with respect to these 45 communities is meaningful.

## Results

### Descriptive summaries

We recorded residency changes for 8,857 men over 35,500.01 person-years, and for 12,158 women over 57,945.35 person-years. The median observation period for men was 3.72 years (IQR = 4.00), while the median observation period for women was 4.41 years (IQR = 5.47). Tables 2, 3, 4 and 5 give cumulative durations of exposure of the repeat-testers stratified by age, calendar year, marital status and education level. The calculation of person-years is based on a random imputation of the seroconversion date between the date of the last negative and first positive test for HIV sero-converters [67], and on the date of the last negative test for those who are censored. We see that longer exposure periods are recorded for younger study participants between 15 and 24 years old. The length of exposure over calendar years remains relatively unchanged between 2005 and 2011, but has a slight tendency to decrease towards 2016. Most repeat-testers were single during the study period, and had different levels of education.

**Table 2:** Length of exposure of the repeat-testers by age stratum.

Age Stratum (years)	Men		Women	
	Person-years	%	Person-years	%
15-19	11,112.86	31.30	12,633.22	21.80
20-24	10,067.54	28.36	12,844.92	22.17
25-29	4,499.06	12.67	6,390.93	11.03
30-34	2,332.67	6.57	4,001.85	6.91
35-39	1,788.91	5.04	4,186.15	7.22
40-44	1,673.96	4.72	5,172.23	8.93
≥45	4,025.02	11.34	12,716.05	21.94
Total	35,500.01	100	5,7945.35	100

**Table 3:** Length of exposure of the repeat-testers by calendar year.

Calendar Year	Men		Women	
	Person-years	%	Person-years	%
2004	1,427.95	4.02	2,194.87	3.79
2005	2,982.77	8.40	4,417.9	7.62
2006	3,471.36	9.78	5,171.38	8.92
2007	3,516.45	9.91	5,385.17	9.29
2008	3,510.61	9.89	5,497.93	9.49
2009	3,378.54	9.52	5,365.29	9.26
2010	3,093.13	8.71	5,076.53	8.76
2011	3,045.45	8.58	5,066.04	8.74
2012	2,652.26	7.47	4,636.20	8.00
2013	2,526.03	7.12	4,458.78	7.69
2014	2,438.65	6.87	4,347.10	7.50
2015	2,248.12	6.33	3,957.17	6.83
2016	1,208.69	3.40	2,370.99	4.09
Total	35,500.01	100	57,945.35	100

**Table 4:** Length of exposure of the repeat-testers by marital status.

Marital Status	Men		Women	
	Person-years	%	Person-years	%
Single	32,770.44	92.31	46,605.16	80.43
Married, monogamous	2,658.74	7.49	9,922.16	17.12
Married, polygamous	70.83	0.20	1,418.03	2.45
Total	35,500.01	100	57,945.35	100

**Table 5:** Length of exposure of the repeat-testers by education level.

Years of Education	Men		Women	
	Person-years	%	Person-years	%
0-5	4,623.64	13.02	11,499.49	19.85
6-9	11,279.37	31.77	14,108.81	24.35
10-11	10,134.68	28.55	16,353.51	28.22
≥12	9,462.32	26.65	15,983.55	27.58
Total	35,500.01	100	57,945.35	100

There were 806 HIV seroconversions in men, and 2,458 HIV seroconversions in women. Table 6 gives seroconversion rates stratified by gender, age (younger or older than 30 years at baseline), and residency outside the rural study area. The largest seroconversion rate 22.47% (95% CI: 21.49-23.45) is for young women who resided in the rural study area for their entire exposure period. The seroconversion rate for young women who resided outside the rural study area is slightly lower: 19.20% (95% CI: 17.88-20.52). The largest seroconversion rate for men is 13.24% (95%CI: 11.76-14.72), and corresponds to the young group that moved outside the rural study area. The seroconversion rate for young men who did not move outside the rural study area is significantly lower: 7.56% (95% CI: 6.88-8.24). Table 6 also shows that the seroconversion rates for both men and women in the older age group are higher for the repeat-testers that moved outside the rural study area as compared to the repeat-testers that did not move outside the rural study area.

**Table 6:** Seroconversion rates and 95% CIs of the repeat-testers.

Gender	Young			
	No		Yes	
	Outside		Outside	
	No	Yes	No	Yes
Men	7.59 (5.91,9.26)	11.06 (7.05,15.07)	7.56 (6.88,8.24)	13.24 (11.76,14.72)
Women	8.60 (7.49,9.71)	12.64 (8.72,16.55)	22.47 (21.49,23.45)	19.20 (17.88,20.52)

The repeat-testers are cross-classified by whether they moved outside the rural study area (*Outside*: Yes/No) and whether they were less than 30 years old at the start of the study (*Young*: Yes/No).

**Fig 1:**
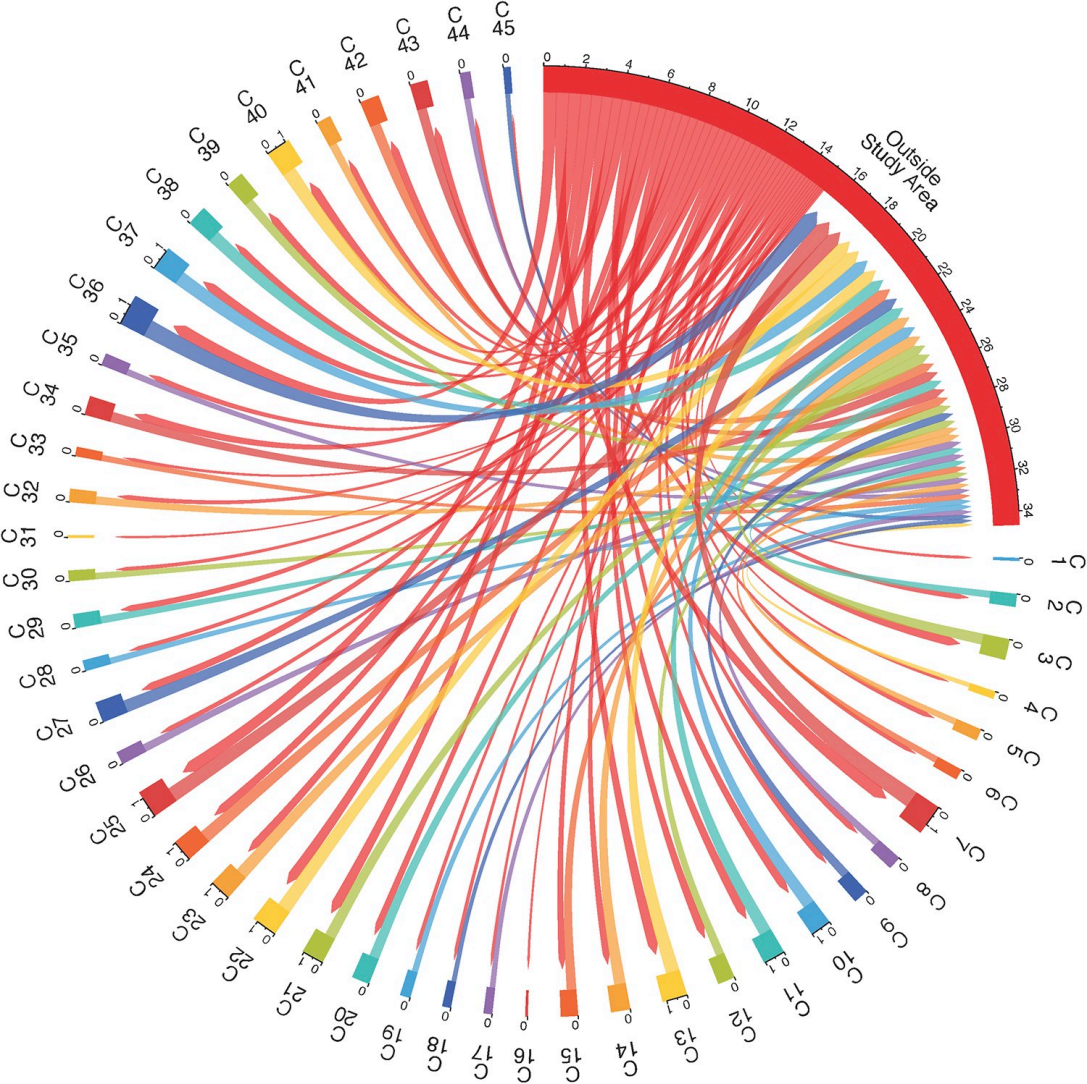
Mobility flows of the repeat-testers who were men. Men moved between the 45 communities labeled C1, C2, …, C45 and locations outside the rural study area. Flows that involve less than 10 men are not depicted.

**Fig 2:**
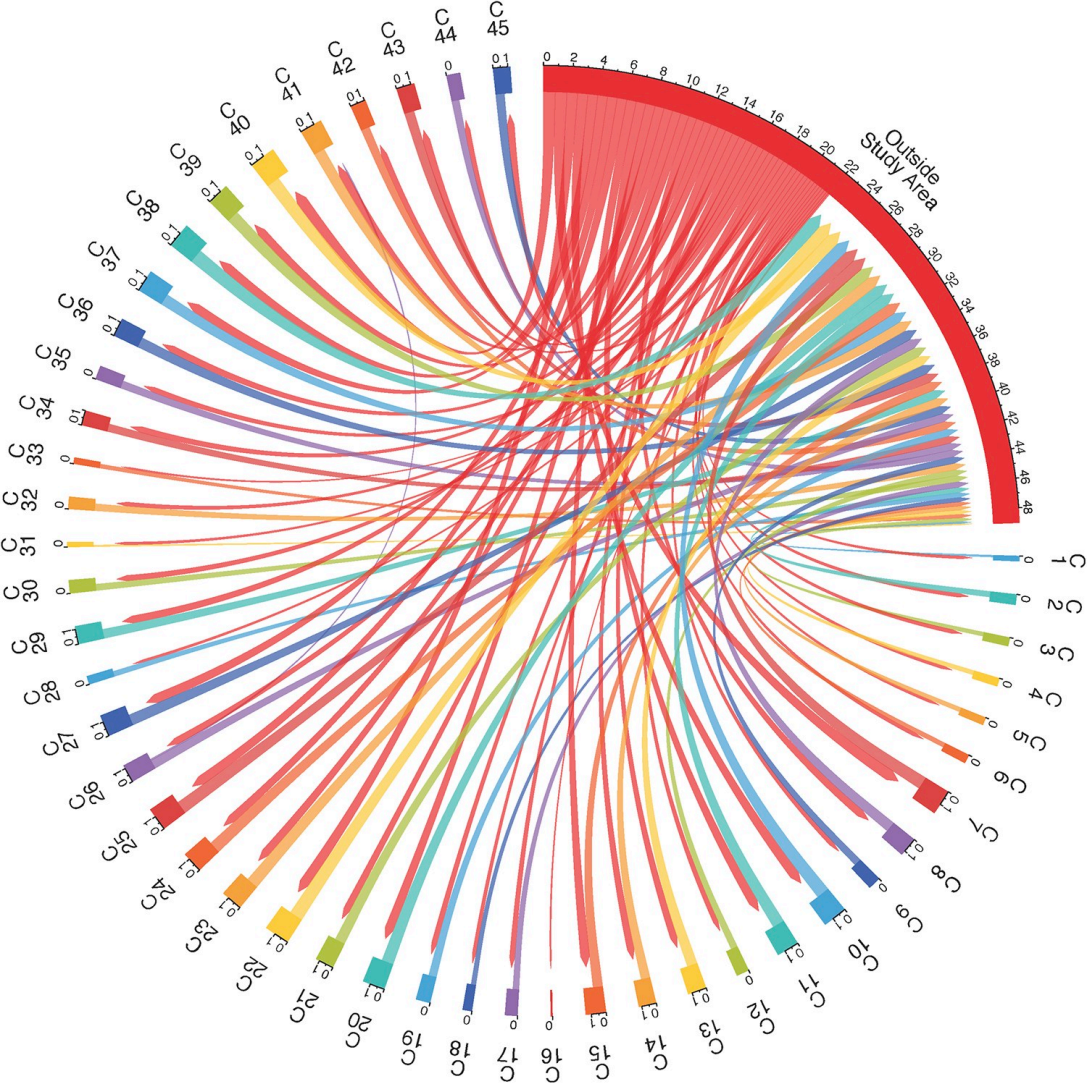
Mobility flows of repeat-testers who were women. Women moved between the 45 communities labeled C1, C2, …, C45 and locations outside the rural study area. Flows that involve less than 10 women are not depicted.

We determined the number of repeat-testers that moved their residence between any two communities, or between a community and a location outside the rural study area. The resulting mobility flow diagrams are shown in Figs 1 and 2. We see that, while men and women move between the 45 communities, substantially larger flows are associated with changes of residencies to and from locations outside the rural study area. Table 7 gives a summary of the frequency of residential movements inside the rural study area, and also between a location outside the rural study area and another location inside or outside the rural study area by age group and gender. Women in the 20-24 age group move outside the rural study area more often than men in the same age group (26.56% vs. 23.31%). Residential movements outside the rural study area become less frequent for women in the 25-29 age group, but are comparable in frequency with residential movements of men in the 25-29 age group. Men in the 30-34 age group move to and from locations outside the rural study area more frequently than women in the 30-34 age group. Residential movements outside the rural study area of women become significantly less frequent in the age groups 35-39, 40-44 and older than 45 as compared to residential movements of men in the same age group. Residential movements inside the rural study area of both men and women are substantially less frequent than residential movements to and from a location outside the rural study area in any age group. However, inside the rural study area, women tend to be more mobile than men in the younger age groups.

**Table 7:** Residential changes of the repeat-testers.

Age	Men		Women	
Stratum (years)	At least once (95% CI)	Two or more times (95% CI)	At least once (95% CI)	Two or more times (95% CI)
*Outside* **residency changes**				
**15–19**	9.92 (9.13,10.71)	2.53 (2.11,2.95)	13.99 (13.13,14.84)	4.05 (3.56,4.54)
**20–24**	23.31 (22.00,24.62)	9.31 (8.41,10.21)	26.56 (25.32,27.80)	12.30 (11.37,13.22)
**25–29**	23.45 (21.46,25.45)	9.65 (8.26,11.04)	22.12 (20.47,23.77)	8.81 (7.69,9.94)
**30–34**	18.49 (16.02,20.95)	7.14 (5.51,8.78)	11.60 (10.02,13.18)	3.87 (2.91,4.82)
**35–39**	16.11 (13.37,18.86)	7.55 (5.57,9.52)	8.82 (7.40,10.25)	3.42 (2.51,4.34)
**40–44**	10.48 (8.08,12.87)	3.97 (2.44,5.49)	5.73 (4.66,6.81)	2.39 (1.69,3.10)
≥**45**	12.75 (10.69,14.80)	6.92 (5.35,8.48)	5.94 (5.02,6.87)	3.09 (2.41,3.77)
**Inside residency changes**				
**15–19**	1.75 (1.40,2.09)	0.25 (0.12,0.39)	3.02 (2.60,3.44)	0.76 (0.55,0.97)
**20–24**	2.47 (1.99,2.95)	0.45 (0.24,0.66)	3.36 (2.86,3.87)	0.84 (0.58,1.10)
**25–29**	1.68 (1.07,2.28)	0.52 (0.18,0.86)	4.53 (3.70,5.36)	1.07 (0.66,1.48)
**30–34**	2.21 (1.27,3.14)	0.53 (0.07,0.98)	3.61 (2.69,4.53)	0.57 (0.20,0.94)
**35–39**	2.47 (1.31,3.63)	0.44 (0.00,0.93)	2.76 (1.94,3.59)	0.33 (0.04,0.62)
**40–44**	2.06 (0.95,3.17)	0.16 (0.00,0.47)	2.45 (1.73,3.16)	0.22 (0,0.44)
≥**45**	1.28 (0.59,1.98)	0.10 (0.00,0.29)	3.21 (2.52,3.91)	0.48 (0.21,0.75)

Percentages of repeat-testers stratified by gender who changed residences between a location outside the rural study area and another location inside or outside the rural study area (outside residency changes, upper panel), or between two locations inside the rural study area (inside residency changes, lower panel)

We remark that residential movements inside the rural study area occur over much smaller distances (mean = 10.44 km, IQR = 9.14 km) compared to residential movements that involve locations outside the rural study area (mean = 128.50 km, IQR = 178.33 km).

### Graphical loglinear models for mobility tables

**Fig 3:**
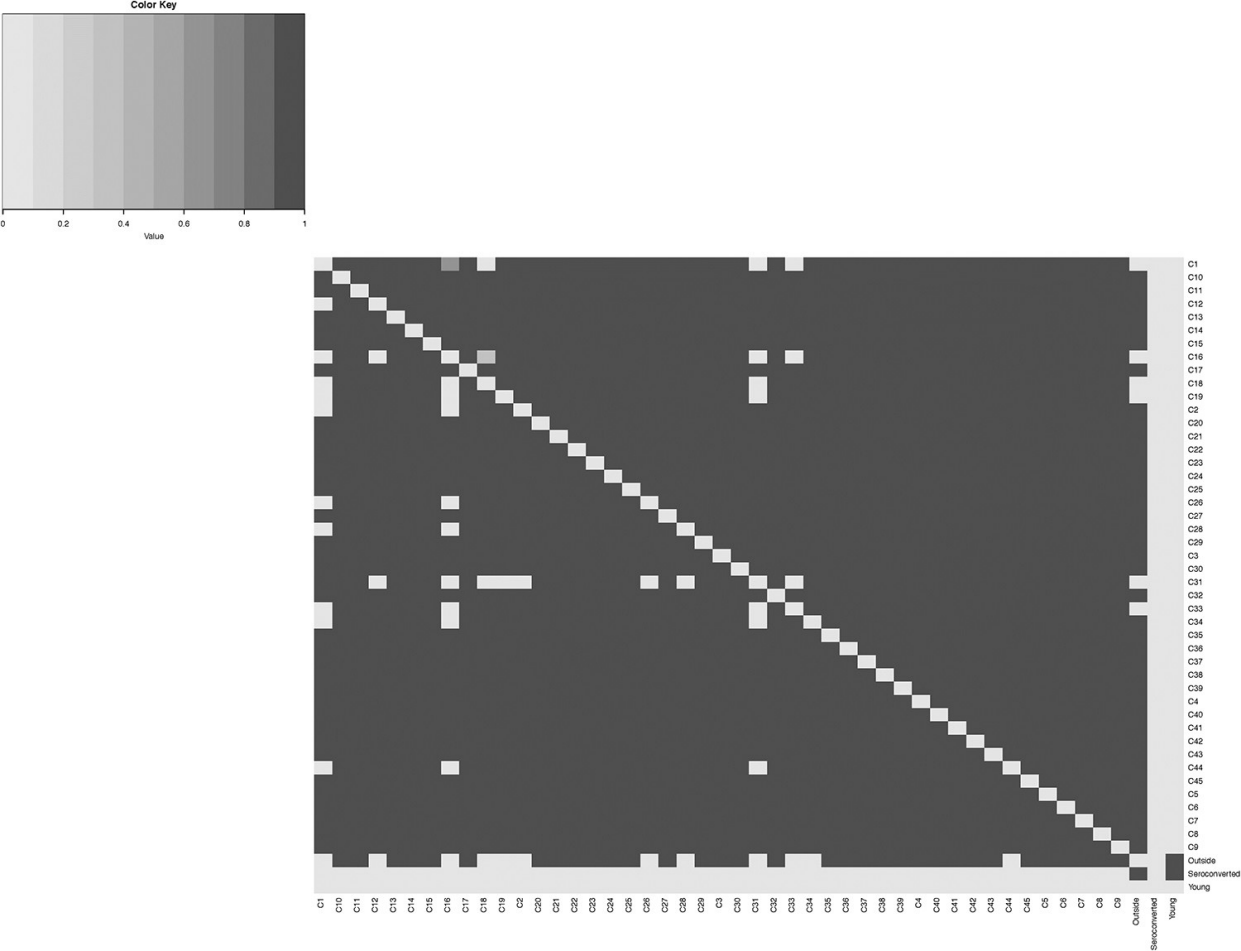
Heatmap of the estimated posterior probabilities of edge inclusion. The matrix entries below the main diagonal show the estimated posterior edge inclusion probabilities for men’s mobility, while the matrix entries above the main diagonal show the estimated posterior edge inclusion probabilities for women’s mobility. Lighter shades of gray indicate smaller (closer to 0) matrix entries, while darker shades of gray indicate larger (closer to 1) matrix entries.

Fig 3 shows a heatmap of the estimated posterior inclusion probabilities of edges connecting the 48 binary variables cross-classified in the mobility tables for men and women. These estimates are based on the 200,000 graphs sampled with the BDMCMC algorithm. For the men’s mobility table, 115 (10.20%) posterior edge inclusion probabilities are 0, and 993 (88.03%) are 1. A number of 18 and 2 edges have estimated posterior inclusion probabilities in (0, 0.5) and [0.5, 1), respectively. For the women’s mobility table, 100 (8.87%) posterior edge inclusion probabilities are 0, and 1,013 (89.80%) are 1. A number of 6 and 9 edges have estimated posterior inclusion probabilities in (0, 0.5), [0.5, 1), respectively. We use the median graph which includes the edges with estimated posterior inclusion probabilities greater than 0.5 as our estimate of the conditional independence graph. The median graph for men’s mobility table has 995 edges, while the median graph for women’s mobility table has 1,022 edges. We refer to these two graphs as men’s and women’s mobility graphs.

The overall structure of the two mobility graphs is remarkably similar. In the men’s mobility graph, the vertex associated with the variable *Outside* is connected with the vertices associated with 33 out of the 45 communities—see the map from Fig E in S1 Supporting Information. The subgraph that involves vertices associated with the 45 communities is dense: it has 1,922 edges—97.07% of the 990 possible edges. In the women’s mobilty graph, the vertex *Outside* is connected with vertices associated with 39 out of 45 communities—see the map from Fig F in S1 Supporting Information. The subgraph associated with the 45 communities is also dense: it has 1,962 edges—99.09% of the 990 possible edges. In both graphs, there is no edge between the vertices associated with variables *Seroconverted* and *Young*, and the community vertices. This implies that, conditional on the variable *Outside*, the variables *Seroconverted* and *Young* are independent of the community variables C1, …, C45 for both men and women.

**Fig 4:**
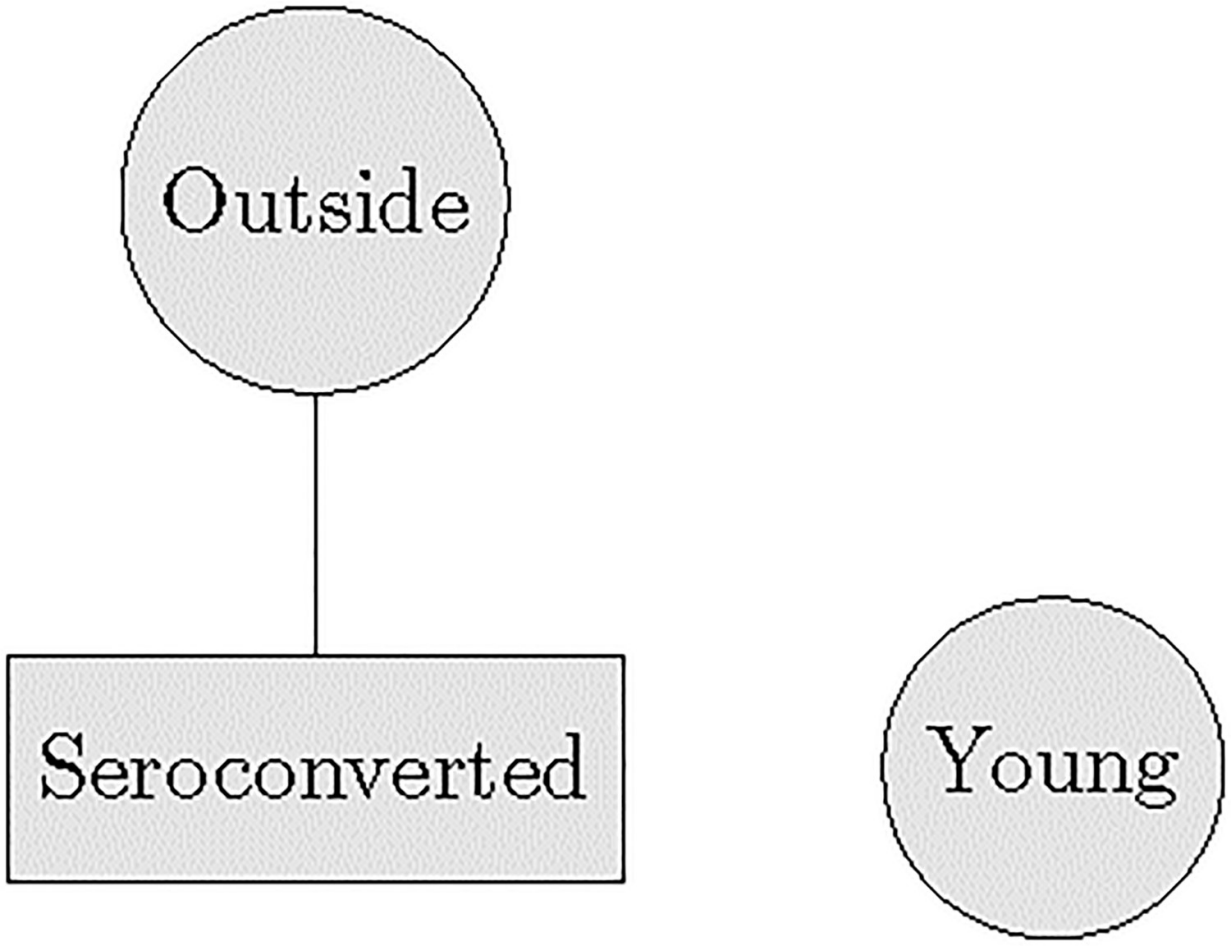
Conditional independence graph for men.

**Fig 5:**
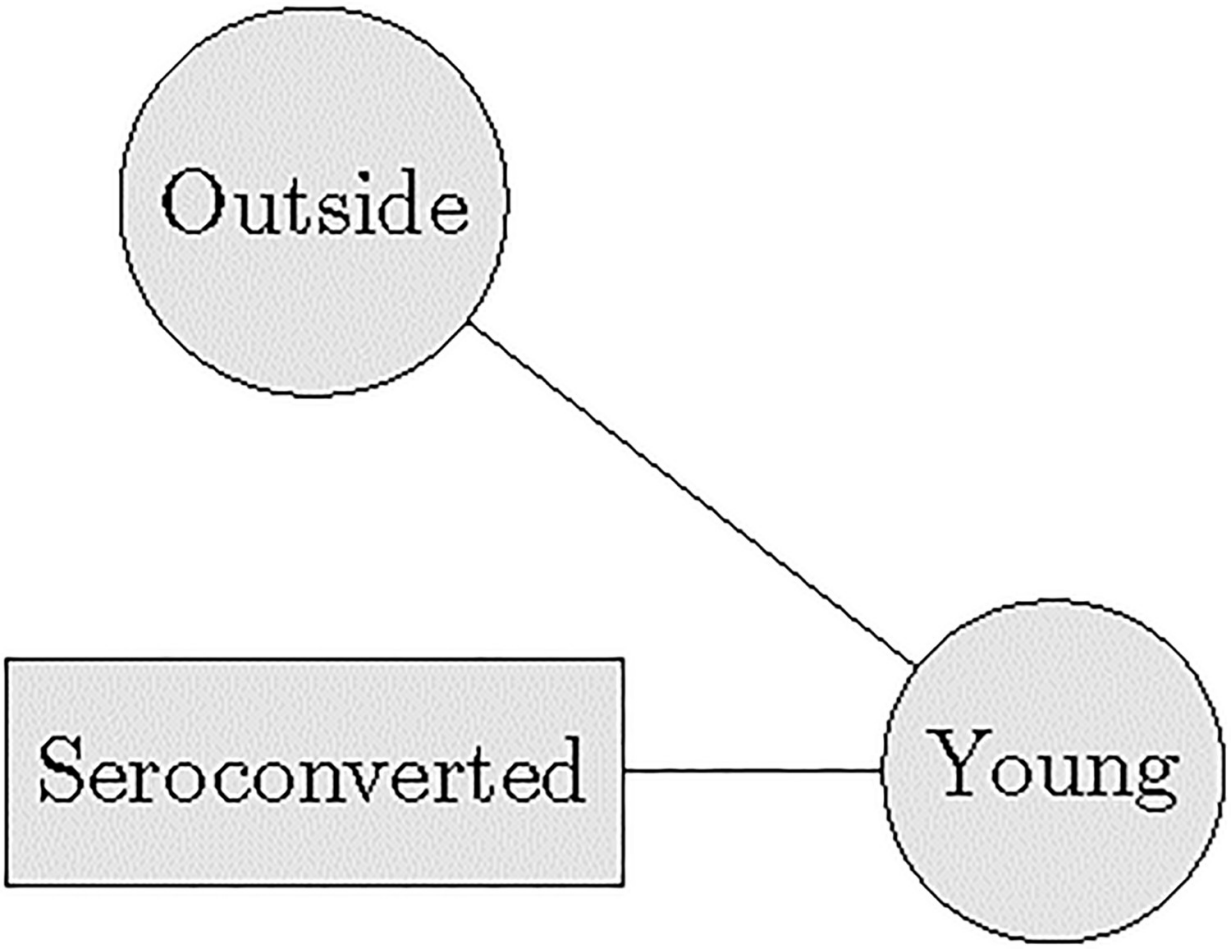
Conditional independence graph for women.

The most relevant differences between the two mobility graphs are related to the edges that link the variables *Outside*, *Seroconverted* and *Young*—see Figs 4 and 5. For men, vertex *Outside* is connected with vertex *Seroconverted*, but the edges between vertices *Outside* and *Young*, and between vertices *Seroconverted* and *Young* are missing. For women, the situation is reversed: the edges between vertices *Outside* and *Young*, and between vertices *Seroconverted* and *Young* are present, but the edge between vertices *Outside* and *Seroconverted* is missing. This has the following implications: (a) for men, variable *Young* is independent of variables *Outside* and *Seroconverted*; (b) for men, only variable *Outside* is predictive of variable *Seroconverted*; (c) for women, variable *Young* is predictive of variable *Seroconverted*; and (d) for women, given variable *Young*, variable *Seroconverted* is independent of variable *Outside*.

The presence of an edge between *Outside* and *Seroconverted* in the subgraph for men means that whether a man moved outside the rural study area is predictive of whether he seroconverts (unadjusted OR = 2.003, 95% CI = [1.718,2.332]). The absence of an edge between *Young* and *Seroconverted* in the same subgraph means that age has less predictive power for the HIV seroconversion of a man given that we know whether this man had a residence outside the rural study area. We point out that this does not imply that age is not a risk factor for HIV acquisition in men. For women, the relative predictive importance of moving outside the rural study area and age is reversed: the edge between *Outside* and *Seroconverted* is missing, while the edge between *Young* and *Seroconverted* is present. Whether a woman is younger than 30 years is predictive of whether she seroconverts (unadjusted OR = 3.091, 95% CI = [2.693,3.561]). However, given that we know the age of a woman, knowing whether she moved outside the rural study area has less predictive power for HIV seroconversion. As such, residential locations seems to matter less for women as a risk factor for HIV acquisition in the presence of age. As an aside, we mention that the presence of an edge that links vertices *Outside* and *Young* in the women’s mobility graph makes sense: women younger than 30 years are more likely to move outside the rural study area (unadjusted OR = 3.176, 95% CI = [2.787,3.633]). This edge is missing in men’s mobility graph because the relationship between variables *Young* and *Outside* is weaker (unadjusted OR = 1.306, 95% CI = [1.124,1.523]).

Since the structure of interactions among variables *Outside*, *Seroconverted* and *Young* is essential for our understanding of the mobility tables, we performed a second statistical analysis of the three-way tables cross-classifying these variables—see Tables A and B in S1 Supporting Information. This time we followed a classical approach to hierarchical loglinear model determination [7, 78] that also solves the structural learning problem, but is conceptually different from the Bayesian approach implemented in the BDMCMC algorithm. We note that this classical approach is suitable for analyzing these two tables because they involve only three variables and they do not contain any counts of 0. However, this approach is not feasible for analyzing the 48-dimensional mobility tables for men and women due to sparsity and the number of variables involved. Specifically, we fitted the eight hierarchical loglinear models that contain main effects for variables *Outside*, *Seroconverted* and *Young*, and also one, two or all three of the pairwise interactions between these variables. The results are presented in Tables C and D in S1 Supporting Information.

For men, the loglinear model that contains interactions between variables *Seroconverted* and *Outside*, and between variables *Outside* and *Young*, and the loglinear model that contains all three pairwise interactions do not fit the data well: the p-values for the likelihood ratio test against the saturated loglinear model are 0.348 and 0.215, respectively. The other six hierarchical models fit the data well at the significance level *α* = 0.05. To select the most relevant model among the remaining six models, we calculated their AIC and BIC. The smallest values for both AIC and BIC are realized for the model that contains the interaction between *Seroconverted* and *Outside*, and no interaction involving variable *Young*. This is precisely the graphical loglinear model we determined before using the BDMCMC algorithm—see Fig 4. For women, the loglinear model that contains all three pairwise interactions does not fit the data well (p-value = 0.264). The other seven hierarchical models fit the data well at the significance level *α* = 0.05. Among these seven models, the model that has the minimum value for both AIC and BIC contains interactions between variables *Outside* and *Young*, and between variables *Seroconverted* and *Young*. As for men, we found the same graphical loglinear model as we did before using the BDMCMC algorithm for the women’s mobility table—see Fig 5.

## Discussion

We proposed a framework for statistical analysis of repeated residential movements. In the first step, residential histories are converted into a mobility matrix that gives the presence and absence patterns from the areas in which study participants have lived. After the inclusion of additional categorical variables of interest, the resulting matrix is converted into a multi-dimensional contingency table called mobility table. The multivariate associations in this table are modeled with graphical loglinear models. The structure of the graphs that characterize these models induces independence or conditional independence relationships among the residential areas and the other categorical variables. This framework is able to explicitly account for individuals that moved across several areas. Existing models for human mobility are able to represent only the movement of an individual from one area to another area without consideration of the areas in which the individual has resided in the past. Our framework also goes beyond those approaches that involve the determination of mobility measures of different kinds; such measures loose an explicit connection with the areas in which residencies were located.

We used this framework to link human mobility and the risk of HIV acquisition based on data from a population-based cohort in a hyper-endemic, rural sub-Saharan African context. The residential locations occupied by every study participant were classified as outside or inside the rural study area. The residential locations inside the rural study area was further classified as belonging to one of 45 non-overlapping communities that fully cover the rural study area. We also included age (younger or older than 30 years at the start of the exposure period) as an additional risk factor for HIV acquisition.

We found that, for both men and women, the majority of residential moves involved a destination outside the rural study area, rather than a destination within the rural study area. Thus, households in the rural study area are typical net-senders of mobile individuals to destinations in the KwaZulu-Natal province, or to other, more distant places throughout South Africa [6]. This circular migration stream effectively links a poor, rural community with more affluent urban centers where many employment opportunities are usually available, and also with other rural areas that offer more specialized types of employment (e.g., mining).

Multivariate predictive relationships are revealed in the mobility graphs for men and women we identified. In both graphs, in order to reach any of the communities vertices C1, C2, …, C45 from the vertex *Seroconverted* by following paths of adjacent edges, we must first pass through the vertex *Outside*. Therefore, once we know whether a man or a woman moved outside the rural study area, knowing which communities inside the rural study area they lived in becomes less relevant for the purpose of predicting whether they seroconverted. For this reason the communities in which an individual resides seem to play a lesser role as risk factors for HIV seroconversion as compared with having a residence outside the rural study area. This finding is surprising because this rural study area has three large irregularly-shaped clusters of new HIV infections near a national road and in a rural node bordering a recent coal mine development [69]. These spatial areas are characterized by HIV incidence rates higher the other surrounding regions. We expected at least some of the communities spanned by these three clusters to be linked by an edge with vertex *Seroconverted*. However, none of these edges are present in the two mobility graphs. Consequently, while the places of residency inside the rural study area certainly play a role in predicting HIV acquisition risk given the significant clustering of HIV infections in this rural community, their predictive power vanishes when taking into account whether a study participant moved outside the rural study area. While this is true for both men and women, the predictive importance of having a residence outside the rural study area differs for men as compared to women. These differences are evidenced in the subgraphs of the two mobility graphs associated with variables *Outside*, *Young* and *Seroconverted*—see Figs 4 and 5.

Our results indicate that, even if the frequency, duration and distance traveled associated with residential moves is similar for men and women who live in this rural study area [6], there must exist key differences between the behavioral processes that lead to HIV seroconversion of mobile men and women. In order to formulate gender-specific combination HIV prevention strategies for high-risk mobile individuals, particularly in the light of attaining the UNAIDS 90-90-90 treatment targets [79], it is of paramount importance to understand these differences with respect to the complex network of structural, biological and socio-demographic factors that characterize places of residency outside the rural study area, and significantly alter the social context of mobile individuals [42].

## Supporting information

S1 Supporting Information Supplementary text and figures. These are additional tables and figures referenced in the paper.
(PDF)

S1 Data Data necessary to replicate the numerical results in the paper. This file contains the mobility matrix for men.
(CSV)

S2 Data Data necessary to replicate the numerical results in the paper. This file contains the mobility matrix for women.
(CSV)

## References

[1] Raymer J, Abel G, Smith PWF. Combining census and registration data to estimate detailed elderly migration flows in England and Wales. Journal of the Royal Statistical Society: Series A (Statistics in Society). 2007;170:891–908. 10.1111/j.1467-985X.2007.00490.x

[2] Smith PWF, Raymer J, Giulietti C. Combining available migration data in England to study economic activity flows over time. Journal of the Royal Statistical Society: Series A (Statistics in Society). 2010;173:733–753. 10.1111/j.1467-985X.2009.00630.x

[3] Raymer J, Wiśniowski A, Forster JJ, Smith PWF, Bijak J. Integrated Modeling of European Migration. Journal of the American Statistical Association. 2013;108:801–819. 10.1080/01621459.2013.789435

[4] Brockmann D, Hufnagel L, Geisel T. The scaling laws of human travel. Nature. 2006;439(7075):462–465. 10.1038/nature04292 16437114

[5] Guerzhoy M, Hertzmann A. Learning Latent Factor Models of Travel Data for Travel Prediction and Analysis. In: Sokolova M, van Beek P, editors. Advances in Artificial Intelligence: 27th Canadian Conference on Artificial Intelligence, Canadian AI 2014, Montréal, QC, Canada, May 6-9, 2014. Proceedings. Cham: Springer International Publishing; 2014. p. 131–142. Available from: 10.1007/978-3-319-06483-3_12.

[6] Dobra A, Bärnighausen T, Vandormael A, Tanser F. Space-time migration patterns and risk of HIV acquisition in rural South Africa. AIDS. 2017;31:137–145. 10.1097/QAD.0000000000001292 27755099 PMC5131684

[7] Bishop YMM, Fienberg SE, Holland PW. Discrete Multivariate Analysis: Theory and Practice. MIT Press, Cambridge, MA; 1975.

[8] Whittaker J. Graphical Models in Applied Multivariate Statistics. John Wiley & Sons; 1990.

[9] Lauritzen SL. Graphical models. vol. 17 Oxford University Press, USA; 1996.

[10] Jones B, Carvalho C, Dobra A, Hans C, Carter C, West M. Experiments in stochastic computation for high-dimensional graphical models. Statistical Science. 2005;20(4):388–400. 10.1214/088342305000000304

[11] Drton M, Maathuis MH. Structure Learning in Graphical Modeling. The Annual Review of Statistics and Its Application. 2017;4:365–393. 10.1146/annurev-statistics-060116-053803

[12] Dobra A, Hans C, Jones B, Nevins JR, Yao G, West M. Sparse graphical models for exploring gene expression data. Journal of Multivariate Analysis. 2004;90:196–212. 10.1016/j.jmva.2004.02.009

[13] Dobra A, Lenkoski A. Copula Gaussian graphical models and their application to modeling functional disability data. The Annals of Applied Statistics. 2011;5(2A):969–993. 10.1214/10-AOAS397

[14] Dobra A, Lenkoski A, Rodriguez A. Bayesian inference for general Gaussian graphical models with application to multivariate lattice data. Journal of the American Statistical Association. 2011;106(496):1418–1433. 10.1198/jasa.2011.tm10465 26924867 PMC4767185

[15] Tanser F, Hosegood V, Bärnighausen T, Herbst K, Nyirenda M, Muhwava W, et al Cohort Profile: Africa Centre Demographic Information System (ACDIS) and population-based HIV survey. International Journal of Epidemiology. 2008;37(5):956–962. 10.1093/ije/dym211 17998242 PMC2557060

[16] Tanser F, Bärnighausen T, Grapsa E, Zaidi J, Newell ML. High coverage of ART associated with decline in risk of HIV acquisition in rural KwaZulu-Natal, South Africa. Science. 2013;339:966–971. 10.1126/science.1228160 23430656 PMC4255272

[17] Quinn TC. Population migration and the spread of types 1 and 2 human immunodeficiency viruses. Proceedings of the National Academy of Sciences. 1994;91:2407–2414. 10.1073/pnas.91.7.2407 PMC43380 8146131

[18] Lurie M. The Epidemiology of Migration and HIV/AIDS in South Africa. Journal of Ethnic and Migration Studies. 2006;32(4):649–666. 10.1080/13691830600610056

[19] Voeten HACM, Vissers DCJ, Gregson S, Zaba B, White RG, de Vlas SJ, et al Strong association between in-migration and HIV prevalence in urban sub-Saharan Africa. Sexually Transmitted Diseases. 2010;37(4):240–243. 19959971 10.1097/OLQ.0b013e3181c3f2d0 PMC3514976

[20] Camlin CS, Hosegood V, Newell ML, McGrath N, Bärnighausen T, Snow RC. Gender, migration and HIV in rural KwaZulu-Natal, South Africa. PLOS ONE. 2010;5:1–10. 10.1371/journal.pone.0011539 PMC2902532 20634965

[21] Deane KD, Parkhurst JO, Johnston D. Linking migration, mobility and HIV. Tropical Medicine and International Health. 2010;15(12):1458–1463. 10.1111/j.1365-3156.2010.02647.x 20958895

[22] Anglewicz P. Migration, marital change, and HIV infection in Malawi. Demography. 2012;49:239–265. 10.1007/s13524-011-0072-x 22109083 PMC3787875

[23] Goldenberg SM, Strathdee SA, Perez-Rosales MD, Sued O. Mobility and HIV in Central America and Mexico: a critical review. Journal of Immigrant and Minority Health. 2012;14(1):48–64. 10.1007/s10903-011-9505-2 21789558

[24] Veary J. Learning from HIV: exploring migration and health in South Africa. Global Public Health. 2012;7(1):58–70. 10.1080/17441692.2010.549494 21360380

[25] Weine SM, Kashuba AB. Labor migration and HIV risk: a systematic review of the literature. AIDS and Behavior. 2012;16:1605–1621. 10.1007/s10461-012-0183-4 22481273 PMC3780780

[26] Cassels S, Jenness SM, Biney AAE, Ampofo WK, Dodoo FNA. Migration, sexual networks, and HIV in Agbogbloshie, Ghana. Demographic Research. 2014;31:861–888. 10.4054/DemRes.2014.31.28 25364298 PMC4214381

[27] Tatem AJ, Hemelaar J, Gray RR, Salemi M. Spatial accessibility and the spread of HIV-1 subtypes and recombinants. AIDS. 2012;26:2351–2360. 10.1097/QAD.0b013e328359a904 22951637

[28] Bärnighausen T, Hosegood V, Timaeus IM, Newell ML. The socioeconomic determinants of HIV incidence: evidence from a longitudinal, population-based study in rural South Africa. AIDS. 2007;21:S29–S38. 10.1097/01.aids.0000300533.59483.95 18040162 PMC2847257

[29] Anglewicz P, Van Landingham M, Manda-Taylor L, Kohler HP. Migration and HIV infection in Malawi. AIDS. 2016;30(13):2099–2105. 10.1097/QAD.0000000000001150 27163708 PMC4965311

[30] Dladla AN, Hiner CA, Qwana E, Lurie M. Speaking to rural women: The sexual partnerships of rural South African women whose partners are migrants. Society in Transition. 2001;32(1):79–82. 10.1080/21528586.2001.10419032

[31] Lurie MN, Williams BG, Zuma K, Mkaya-Mwamburi D, Garnett GP, Sweat MD, et al Who infects whom? HIV-1 concordance and discordance among migrant and non-migrant couples in South Africa. AIDS. 2003;17:2245–2252. 14523282 10.1097/00002030-200310170-00013

[32] Collinson MA. Striving against adversity: the dynamics of migration, health and poverty in rural South Africa. Global Health Action. 2010;3 10.3402/gha.v3i0.5080 PMC2882287 20531981

[33] Hunter M. The changing political economy of sex in South Africa: the significance of unemployment and inequalities to the scale of the AIDS pandemic. Social Science & Medicine. 2007;64:689–700. 10.1016/j.socscimed.2006.09.015 17097204

[34] Hargreaves JR, Bonell CP, Morison LA, Kim JC, Phetla G, Porter JDH, et al Explaining continued high HIV prevalence in South Africa: socioeconomic factors, HIV incidence and sexual behaviour change among a rural cohort, 2001–2004. AIDS. 2007;21:S39–S48. 10.1097/01.aids.0000300534.97601.d6 18040163

[35] Munyewende P, Rispel LC, Harris B, Chersich M. Exploring perceptions of HIV risk and health service access among Zimbabwean migrant women in Johannesburg: a gap in health policy in South Africa? Journal of Public Health Policy. 2011;32:S152–S161. 10.1057/jphp.2011.36 21730988

[36] Giorgio M, Townsend L, Zembe Y, Cheyip M, Guttmacher S, Carter R, et al HIV prevalence and risk factors among male foreign migrants in Cape Town, South Africa. AIDS and Behavior. 2014;18:2020–2029. 10.1007/s10461-014-0784-1 27557987 PMC6004499

[37] Colebunders R, Kenyon C. Behaviour, not mobility, is a risk factor for HIV. The Lancet HIV. 2015;2:e223–e224. 10.1016/S2352-3018(15)00057-0 26423190

[38] McGrath N, Eaton JW, Newell ML, Hosegood V. Migration, sexual behaviour, and HIV risk: a general population cohort in rural South Africa. The Lancet HIV. 2015;2:e252–e259. 10.1016/S2352-3018(15)00045-4 26280016 PMC4533230

[39] Vandormael A, Newell ML, Bärnighausen T, Tanser F. Use of antiretroviral therapy in households and risk of HIV acquisition in rural KwaZulu-Natal, South Africa, 2004–12: a prospective cohort study. The Lancet Global Health. 2014;2:e209–e215. 10.1016/S2214-109X(14)70018-X 24782953 PMC3986029

[40] Vearey J, Palmary I, Thomas L, Nunez L, Drimie S. Urban health in Johannesburg: the importance of place in understanding intra-urban inequalities in a context of migration and HIV. Health & Place. 2010;16:694–702. 10.1016/j.healthplace.2010.02.007 20400354

[41] Tanser F, de Oliveira T, Maheu-Giroux M, Bärnighausen T. Concentrated HIV sub-epidemics in generalized epidemic settings. Current Opinion in HIV and AIDS. 2014;9:115–125. 10.1097/COH.0000000000000034 24356328 PMC4228373

[42] Tomita A, Vandormael AM, Bärnighausen T, de Oliveira T, Tanser F. Social disequilibrium and the risk of HIV acquisition: a multilevel study in rural KwaZulu-Natal Province, South Africa. Journal of Acquired Immune Deficiency Syndrome. 2017;75:164–174. 10.1097/QAI.0000000000001349 PMC5429974 28291049

[43] Tanser F, Bärnighausen T, Hund L, Garnett GP, McGrath N, Newell ML. Effect of concurrent sexual partnerships on rate of new HIV infections in a high-prevalence, rural South African population: a cohort study. The Lancet. 2011;378:247–255. 10.1016/S0140-6736(11)60779-4 PMC3141142 21763937

[44] Tanser F, Vandormael A, Cuadros D, Phillips AN, de Oliveira T, Tomita A, et al Effect of population viral load on prospective HIV incidence in a hyperendemic rural African community. Science Translational Medicine. 2017;9 10.1126/scitranslmed.aam8012 29237762 PMC6435884

[45] Lurie MN, Williams BG, Zuma K, Mkaya-Mwamburi D, Garnett GP, Sturm AW, et al The impact of migration on HIV-1 transmission in South Africa: a study of migrant and nonmigrant men and their partners. Sexually Transmitted Diseases. 2003;30:149–156. 12567174 10.1097/00007435-200302000-00011

[46] Cassels S, Jenness SM, Khanna AS. Conceptual framework and research methods for migration and HIV transmission dynamics. AIDS and Behavior. 2014;18:2302–2313. 10.1007/s10461-013-0665-z 24257897 PMC4029933

[47] Lurie MN, Williams BG. Migration and health in Southern Africa: 100 years and still circulating. Health Psychology and Behavioral Medicine: An Open Access Journal. 2014;2:34–40. 10.1080/21642850.2013.866898 PMC3956074 24653964

[48] National Department of Health of South Africa. The 2011 National Antenatal Sentinel HIV & syphilus prevalence survey in South Africa; 2012. Available online at https://www.health-e.org.za.

[49] Statistics South Africa. Mid-year population estimates, South Africa; 2004. Available online at http://www.statssa.gov.za/.

[50] Jochelson K, Mothibeli M, Leger JP. Human Immunodeficiency Virus and Migrant Labor in South Africa. International Journal of Health Services. 1991;21:157–173. 10.2190/11UE-L88J-46HN-HR0K 2004869

[51] Hargrove J. Migration, mines and mores: the HIV epidemic in southern Africa. South African Journal of Science. 2008;104:53–61.

[52] Lurie M, Harrison A, Wilkinson D, Karim SA. Circular migration and sexual networking in rural KwaZulu-Natal: implications for the spread of HIV and other sexually transmitted diseases. Health Transition Review. 1997;7:17–27. 10175971

[53] Rasmussen DA, Wilkinson E, Vandormael A, Tanser F, Pillay D, Stadler T, et al Tracking external introductions of HIV using phylodynamics reveals a major source of infections in rural KwaZulu-Natal, South Africa. Virus Evolution. 2018;4:vey037 10.1093/ve/vey037 30555720 PMC6290119

[54] Posel D. Influx control and urban labour markets in the 1950s In: Bonner PL, Delius P, Posel D, editors. Apartheid’s genesis, 1935-1962. Ravan Press of South Africa; 1993.

[55] Preston-Whyte E. Women who are not married: Fertility, ‘illegitimacy’, and the nature of households and domestic groups among single African women in Durban. South African Journal of Sociology. 1993;24:63–71. 10.1080/02580144.1993.10432905

[56] Hosegood V, McGrath N, Moultrie T. Dispensing with marriage: marital and partnership trends in rural KwaZulu-Natal, South Africa 2000-2006. Demographic Research. 2009;20:279–312. 10.4054/DemRes.2009.20.13 25729322 PMC4340557

[57] Moore H. Households and gender in a South African bantustan. African Studies. 1994;53:137–142. 10.1080/00020189408707792

[58] Butler A. South Africa’s HIV/AIDS policy, 1994-2004: How can it be explained? African Affairs. 2005;104:591–614. 10.1093/afraf/adi036

[59] Nattrass N. Mortal Combat: AIDS denialism and the struggle for antiretrovirals in South Africa. University of KwaZulu-Natal Press; 2007.

[60] Vandormael A. The TAC’s “Intellectual Campaign” (2000–2004): Social Movements and Epistemic Communities. Politikon. 2007;34:217–233. 10.1080/02589340701725306

[61] Forsyth B, Vandormael A, Kershaw T, Grobbelaar J. The political context of AIDS-related stigma and knowledge in a South African township community. SAHARA J: Journal of Social Aspects of HIV/AIDS Research Alliance. 2008;5:74–82. 10.1080/17290376.2008.9724904 PMC4239696 18709210

[62] Department of Health. The South African antiretroviral treatment guidelines: 2010. Pretoria; 2010.

[63] Shisana O, Rehle T, Simbayi LC, Zuma K, Jooste S, Zungu N, et al. South African National HIV Prevalence, Incidence and Behaviour Survey, 2012. Human Sciences Resource Council Press; 2014. 10.2989/16085906.2016.1153491 27002359

[64] Human Sciences Research Council. The Fifth South African National HIV Prevalence, Incidence, Behaviour and Communication Survey, 2017; 2018.

[65] Muhwava W, Hosegood V, Nyirenda M, Herbst K, Newell ML. Levels and determinants of migration in rural KwaZulu-Natal, South Africa. African Population Studies. 2010;24:259–280.

[66] Strode A, Slack C, Essack Z. Child consent in South African law: Implications for researchers, service providers and policy-makers. South African Medical Journal. 2010;100:247–249. 10.7196/SAMJ.3609 20459973

[67] Vandormael A, Dobra A, Bärnighausen T, de Oliveira T, Tanser F. Incidence rate estimation, periodic testing and the limitations of the mid-point imputation approach. International Journal of Epidemiology. 2018;47:236–245. 10.1093/ije/dyx134 29024978 PMC5837439

[68] Tanser F, Bärnighausen T, Cooke GS, Newell ML. Localized spatial clustering of HIV infections in a widely disseminated rural South African epidemic. International Journal of Epidemiology. 2009;38:1008–1016. 10.1093/ije/dyp148 19261659 PMC2720393

[69] Tanser F, Bärnighausen T, Dobra A, Sartorius B. Identifying “corridors of HIV transmission” in a severely affected rural South African population: a case for a shift toward targeted prevention strategies. International Journal of Epidemiology. 2018;47:537–549. 10.1093/ije/dyx257 29300904 PMC5913614

[70] Dobra A, Mohammadi R. Loglinear model selection and human mobility. Annals of Applied Statistics. 2018;12:815–845. 10.1214/18-AOAS1164

[71] Preston CJ. Spatial birth-and-death processes. Bulletin of the International Statistical Institute. 1975;46:371–391.

[72] Mohammadi R, Wit EC, Dobra A. BDgraph: Bayesian Structure Learning in Graphical Models using Birth-Death MCMC; 2018. Available from: http://CRAN.R-project.org/package=BDgraph.

[73] Mohammadi A, Dobra A. The R package BDgraph for Bayesian structure learning in graphical models. ISBA Bulletin. 2017;4:11–16.

[74] R Core Team. R: A Language and Environment for Statistical Computing; 2018. Available from: https://www.R-project.org/.

[75] Dobra A, Lenkoski A, Rodriguez A. Bayesian inference for general Gaussian graphical models with application to multivariate lattice data. Journal of the American Statistical Association. 2011;106:1418–1433. 10.1198/jasa.2011.tm10465 26924867 PMC4767185

[76] Putnam SH, Chung SH. Effects of spatial systems design on spatial interaction models. 1: The spatial definition problem. Environment and Planning A. 1989;21:27–46. 10.1068/a210027

[77] Fotheringham AS, Wong DWS. The multivariate areal unit problem in multivariate statistical analysis. Environment and Planning A. 1991;23:1025–1044. 10.1068/a231025

[78] Fienberg SE. The Analysis of Cross-Classified Categorical Data. MIT Press, Cambridge, MA; 1980.

[79] UNAIDS. 90–90–90—An ambitious treatment target to help end the AIDS epidemic; 2014. UNAIDS.

