# Supplementary material for: A method for statistical analysis of repeated residential movements to link human mobility and HIV acquisition
Source: PLoS One. 2019 Jun 5;14(6):e0217284. doi: 10.1371/journal.pone.0217284 (PMC6550382; doi:10.1371/journal.pone.0217284)
Supplement: S1 Supporting Information — These are additional tables and figures referenced in the paper. (PDF) [file pone.0217284.s001.pdf]

SUPPORTING INFORMATION FOR "A METHOD FOR STATISTICAL ANALYSIS OF REPEATED RESIDENTIAL MOVEMENTS TO LINK HUMAN MOBILITY AND HIV ACQUISITION"

ADRIAN DOBRA, TILL BÄRNIGHAUSEN, ALAIN VANDORMAEL AND FRANK TANSER

Contents

List of Figures 2
List of Tables 2

List of Figures

A Annual consent rates for the study duration. 3

B Annual consent rates by age group and gender for the study duration. 4

C Convergence plot of the BDMCMC algorithm showing the estimated posterior inclusion probabilities of edges in graphs associated with men's mobility. 14

D Convergence plot of the BDMCMC algorithm showing the estimated posterior inclusion probabilities of edges in graphs associated with women's mobility. 15

E Map of the communities that are linked with an edge with the vertex *Outside* in the estimated conditional independence graph for men's mobility. A number of 33 communities (gray) are linked with *Outside*, while 12 communities (white) are not linked with an edge with *Outside*. 30

F Map of the communities that are linked with an edge with the vertex *Outside* in the estimated conditional independence graph for women's mobility. A number of 39 communities (gray) are linked with *Outside*, while 6 communities (white) are not linked with an edge with *Outside*. 31

List of Tables

A Cross-classification of 8,857 men that participated in the study by their HIV seroconversion status (*Seroconverted*: Yes/No), whether they moved outside the study area (*Outside*: Yes/No) and whether they were less than 30 years old at the start of the study (*Young*: Yes/No). 4

E Cells with positive counts in the 48-dimensional dichotomous mobility table for men. Each row in the table is associated with one positive cell count. The first column gives the index of each count when the cells are ordered in decreasing order of their counts. The second column gives the names of the variables that take value "yes" for that count; the variables whose name do not appear take value "no". The third column gives the value of the cell count. 4

B Cross-classification of 12,158 women that participated in the study by their HIV seroconversion status (*Seroconverted*: Yes/No), whether they moved outside the study area (*Outside*: Yes/No) and whether they were less than 30 years old at the start of the study (*Young*: Yes/No). 14

F Cells with positive counts in the 48-dimensional dichotomous mobility table for women. Each row in the table is associated with one positive cell count. The first column gives the index of each count when the cells are ordered in decreasing order of their counts. The second column gives the names of the variables that take value "yes" for that count; the variables whose name do not appear take value "no". The third column gives the value of the cell count. 15

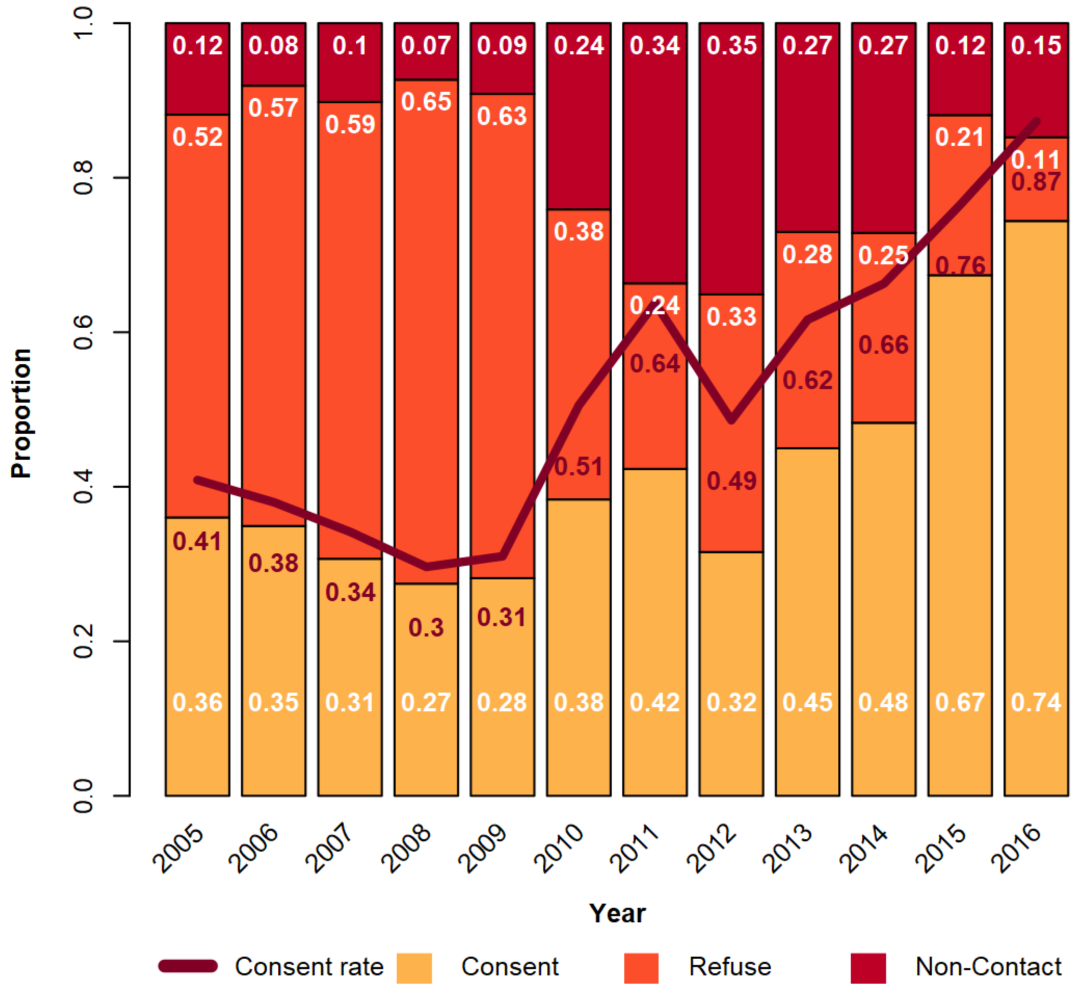

Fig. A. Annual consent rates for the study duration.

C The fit of three-way loglinear models for the cross-classification of 8,857 men from Table A. "Dev" stands for deviance, and "DF" stands for degrees of freedom. The loglinear models are specified by their maximal interaction terms. 32

D The fit of three-way loglinear models for the cross-classification of 12,158 women from Table B. "Dev" stands for deviance, and "DF" stands for degrees of freedom. The loglinear models are specified by their maximal interaction terms. 33

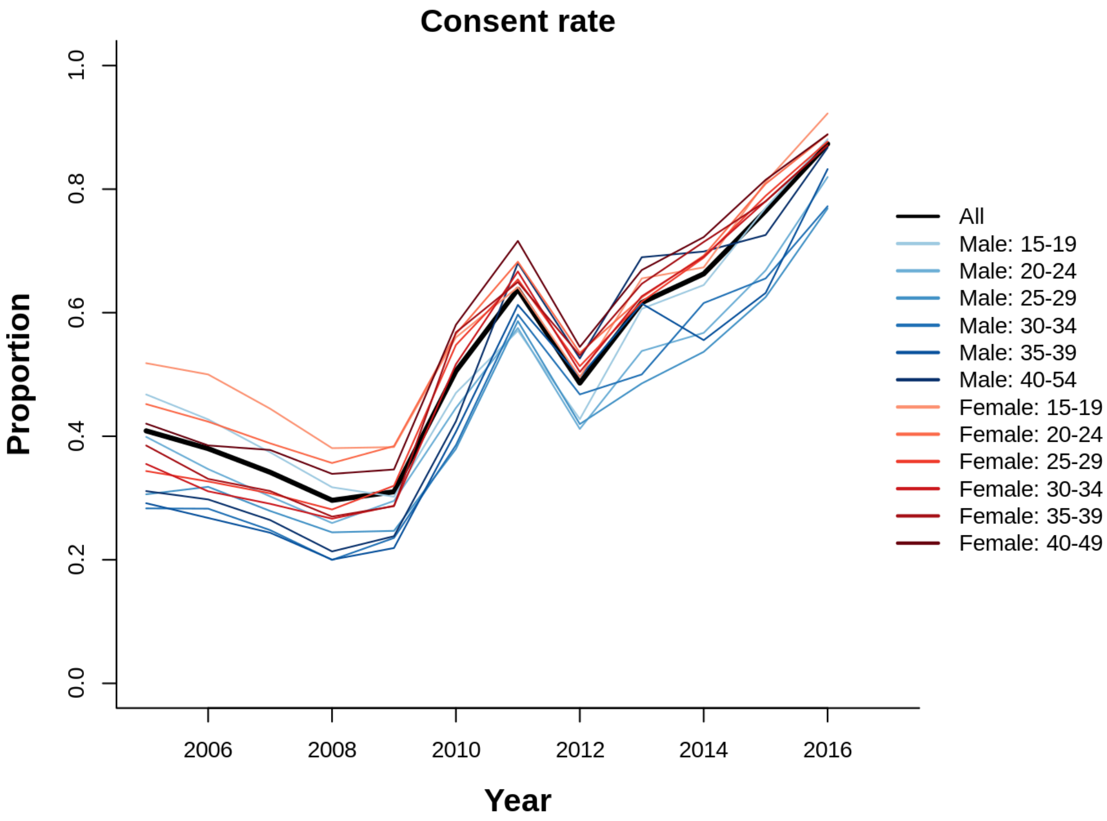

Fig. B. Annual consent rates by age group and gender for the study duration.

	**Young**			
	No		Yes	
	**Outside**		**Outside**	
**Seroconverted**	No	Yes	No	Yes
No	889	209	5368	1585
Yes	73	26	439	268

Table A. Cross-classification of 8,857 men that participated in the study by their HIV seroconversion status (*Seroconverted*: Yes/No), whether they moved outside the study area (*Outside*: Yes/No) and whether they were less than 30 years old at the start of the study (*Young*: Yes/No).

Table E: Cells with positive counts in the 48-dimensional dichotomous mobility table for men. Each row in the table is associated with one positive cell count. The first column gives the index of each count when the cells are ordered in decreasing order of their counts. The second column gives the names of the variables that take value “yes” for that count; the variables whose name do not appear take value “no”. The third column gives the value of the cell count.

Id.	Variables with level “yes”	Count
1	C7, *Young*	192

Table E – continued from previous page

Id.	Variables with level "yes"	Count
2	C37, *Young*	186
3	C40, *Young*	180
4	C39, *Young*	177
5	C22, *Young*	168
6	C36, *Young*	160
7	C25, *Young*	159
8	C20, *Young*	159
9	C10, *Young*	158
10	C24, *Young*	157
11	C38, *Young*	156
12	*Outside*, *Young*	154
13	C21, *Young*	149
14	C23, *Young*	147
15	C27, *Young*	145
16	C13, *Young*	144
17	C42, *Young*	136
18	C11, *Young*	131
19	C35, *Young*	127
20	C15, *Young*	127
21	C14, *Young*	126
22	C43, *Young*	125
23	C5, *Young*	123
24	C34, *Young*	119
25	C4, *Young*	115
26	C9, *Young*	112
27	C3, *Young*	111
28	C8, *Young*	110
29	C17, *Young*	107
30	C32, *Young*	101
31	C41, *Young*	100
32	C29, *Young*	96
33	C6, *Young*	93
34	C19, *Young*	93
35	C30, *Young*	92
36	C45, *Young*	89
37	C12, *Young*	87
38	C2, *Young*	84
39	C44, *Young*	71
40	C28, *Young*	69
41	C18, *Young*	63
42	C26, *Young*	59
43	C7, *Outside*, *Young*	55
44	C25, *Outside*, *Young*	51
45	C13, *Outside*, *Young*	49
46	C36, *Outside*, *Young*	46
47	C37, *Outside*, *Young*	46
48	C22, *Outside*, *Young*	46
49	C24, *Outside*, *Young*	45
50	C22	44
51	C40, *Outside*, *Young*	42
52	C27, *Outside*, *Young*	42
53	C42, *Outside*, *Young*	40
54	C33, *Young*	40
55	C38, *Outside*, *Young*	40
56	C10, *Outside*, *Young*	39
57	C43, *Outside*, *Young*	39
58	*Outside*	39
59	C25	38
60	C39, *Outside*, *Young*	36
61	C20, *Outside*, *Young*	35
62	C3, *Outside*, *Young*	35
63	C11, *Outside*, *Young*	33
64	C12, *Outside*, *Young*	33
65	C23, *Outside*, *Young*	33
66	C14, *Outside*, *Young*	31
67	C16, *Young*	31
68	C39	30
69	C21, *Outside*, *Young*	30

Table E – continued from previous page

Id.	Variables with level "yes"	Count
70	C15, *Outside*, *Young*	30
71	C37	29
72	C31, *Young*	29
73	C40	28
74	C24	28
75	C29, *Outside*, *Young*	28
76	C41, *Outside*, *Young*	28
77	C7	27
78	C11	26
79	C8, *Outside*, *Young*	26
80	C30, *Outside*, *Young*	26
81	C32, *Outside*, *Young*	26
82	C20	26
83	C26, *Outside*, *Young*	26
84	C21	25
85	*Outside*, *Seroconverted*, *Young*	25
86	C17	25
87	C14	24
88	C15	24
89	C1, *Young*	24
90	C44, *Outside*, *Young*	24
91	C43	23
92	C35, *Outside*, *Young*	23
93	C32	23
94	C20, *Seroconverted*, *Young*	23
95	C28, *Outside*, *Young*	22
96	C34, *Outside*, *Young*	22
97	C9, *Outside*, *Young*	22
98	C4	21
99	C36	21
100	C30	20
101	C5, *Outside*, *Young*	20
102	C5, *Seroconverted*, *Young*	20
103	C34	20
104	C19	19
105	C18, *Outside*, *Young*	19
106	C42	19
107	C33, *Outside*, *Young*	19
108	C23	19
109	C41	19
110	C2, *Outside*, *Young*	19
111	C22, *Seroconverted*, *Young*	19
112	C6, *Outside*, *Young*	19
113	C21, *Seroconverted*, *Young*	19
114	C3	19
115	C17, *Outside*, *Young*	19
116	C17, *Seroconverted*, *Young*	19
117	C28	18
118	C3, *Seroconverted*, *Young*	18
119	C44	18
120	C10	17
121	C7, *Seroconverted*, *Young*	17
122	C45	17
123	C38	17
124	C25, *Seroconverted*, *Young*	17
125	C45, *Outside*, *Young*	17
126	C13	17
127	C35	16
128	C4, *Outside*, *Young*	16
129	C5	16
130	C2, *Seroconverted*, *Young*	16
131	C9	16
132	C19, *Outside*, *Young*	16
133	C37, *Seroconverted*, *Young*	16
134	C26	16
135	C18	15
136	C7, *Outside*, *Seroconverted*, *Young*	14
137	C29	14

Table E – continued from previous page

Id.	Variables with level "yes"	Count
138	C6	14
139	C4,*Seroconverted*, *Young*	14
140	C27	14
141	C36, *Outside*,*Seroconverted*, *Young*	14
142	C10, *Outside*,*Seroconverted*, *Young*	13
143	C40,*Seroconverted*, *Young*	12
144	C34,*Seroconverted*, *Young*	12
145	C8	12
146	C8,*Seroconverted*, *Young*	12
147	C11,*Seroconverted*, *Young*	12
148	C2	12
149	C35,*Seroconverted*, *Young*	11
150	C36,*Seroconverted*, *Young*	11
151	C32,*Seroconverted*, *Young*	11
152	C6,*Seroconverted*, *Young*	11
153	C40, *Outside*,*Seroconverted*, *Young*	10
154	C39,*Seroconverted*, *Young*	10
155	C32, *Outside*,*Seroconverted*, *Young*	10
156	C12	10
157	C23, *Outside*	9
158	C27, *Outside*,*Seroconverted*, *Young*	9
159	C21, *Outside*,*Seroconverted*, *Young*	9
160	C11, *Outside*,*Seroconverted*, *Young*	9
161	C2, *Outside*,*Seroconverted*, *Young*	9
162	C22, *Outside*,*Seroconverted*, *Young*	8
163	C37, *Outside*	8
164	C15, *Outside*,*Seroconverted*, *Young*	8
165	C42,*Seroconverted*, *Young*	8
166	C14, *Outside*	8
167	C19,*Seroconverted*, *Young*	8
168	C38, *Outside*	8
169	C29,*Seroconverted*, *Young*	8
170	C14, *Outside*,*Seroconverted*, *Young*	8
171	C23,*Seroconverted*, *Young*	8
172	C38,*Seroconverted*, *Young*	7
173	C13, *Outside*	7
174	C33,*Seroconverted*, *Young*	7
175	C33	7
176	C15,*Seroconverted*, *Young*	7
177	C22,*Seroconverted*	7
178	C34, *Outside*,*Seroconverted*, *Young*	6
179	C45, *Outside*,*Seroconverted*, *Young*	6
180	C10,*Seroconverted*, *Young*	6
181	C22, *Outside*	6
182	C25, *Outside*	6
183	C13,*Seroconverted*, *Young*	6
184	C29, *Outside*,*Seroconverted*, *Young*	6
185	C34, *Outside*	6
186	C9, *Outside*	6
187	C39, *Outside*,*Seroconverted*, *Young*	6
188	C24, *Outside*	5
189	C37, *Outside*,*Seroconverted*, *Young*	5
190	C38, *Outside*,*Seroconverted*, *Young*	5
191	C9,*Seroconverted*, *Young*	5
192	C23, *Outside*,*Seroconverted*, *Young*	5
193	C5, *Outside*,*Seroconverted*, *Young*	5
194	C11, *Outside*	5
195	C25, *Outside*,*Seroconverted*, *Young*	5
196	C24,*Seroconverted*, *Young*	5
197	*Outside*,*Seroconverted*	5
198	C34,*Seroconverted*	5
199	C36, *Outside*	5
200	C1	5
201	C43,*Seroconverted*, *Young*	4
202	C13,C43, *Young*	4
203	C41,*Seroconverted*, *Young*	4
204	C20, *Outside*	4

Table E – continued from previous page

Id.	Variables with level "yes"	Count
205	C27, *Outside*	4
206	C15, *Outside*	4
207	C37, *Seroconverted*	4
208	C7, *Outside*	4
209	C24, *Outside*, *Seroconverted*, *Young*	4
210	C8, *Outside*	4
211	C13, *Outside*, *Seroconverted*, *Young*	4
212	C31, *Seroconverted*, *Young*	4
213	C30, *Seroconverted*, *Young*	4
214	C28, *Seroconverted*, *Young*	4
215	C2, *Outside*, *Seroconverted*	4
216	C12, *Seroconverted*, *Young*	4
217	C10, *Outside*	4
218	C20, *Outside*, *Seroconverted*, *Young*	4
219	C1, *Outside*, *Young*	4
220	C44, *Seroconverted*, *Young*	4
221	C12, *Outside*, *Seroconverted*, *Young*	4
222	C42, *Outside*	4
223	C40, *Outside*	4
224	C3, *Outside*, *Seroconverted*, *Young*	4
225	C6, *Outside*	4
226	C41, *Outside*	4
227	C16, *Seroconverted*, *Young*	4
228	C16	4
229	C26,C41, *Young*	3
230	C27, *Seroconverted*, *Young*	3
231	C43, *Outside*	3
232	C26, *Outside*	3
233	C16,C31, *Young*	3
234	C35, *Outside*	3
235	C5, *Seroconverted*	3
236	C7, *Seroconverted*	3
237	C35, *Seroconverted*	3
238	C9, *Outside*, *Seroconverted*, *Young*	3
239	C21, *Seroconverted*	3
240	C21,C7, *Young*	3
241	C4, *Outside*, *Seroconverted*, *Young*	3
242	C12, *Outside*	3
243	C28, *Outside*, *Seroconverted*, *Young*	3
244	C36, *Seroconverted*	3
245	C8, *Outside*, *Seroconverted*, *Young*	3
246	C42, *Seroconverted*	3
247	C42, *Outside*, *Seroconverted*, *Young*	3
248	C17,C2, *Outside*, *Young*	3
249	C14, *Seroconverted*, *Young*	3
250	C29, *Outside*	3
251	C21, *Outside*	3
252	C28, *Outside*	3
253	C6, *Outside*, *Seroconverted*, *Young*	3
254	C35, *Outside*, *Seroconverted*, *Young*	3
255	C39,C40, *Young*	3
256	C21,C35, *Young*	3
257	C19, *Outside*	3
258	C31, *Outside*, *Young*	3
259	C39, *Outside*	2
260	C10, *Seroconverted*	2
261	C20,C33, *Outside*, *Young*	2
262	C25, *Seroconverted*	2
263	C9, *Seroconverted*	2
264	C43, *Seroconverted*	2
265	C40, *Seroconverted*	2
266	C18, *Seroconverted*, *Young*	2
267	C23, *Seroconverted*	2
268	C14,C43, *Outside*, *Young*	2
269	C13, *Outside*, *Seroconverted*	2
270	C16, *Outside*, *Young*	2
271	C30, *Seroconverted*	2

Table E – continued from previous page

Id.	Variables with level "yes"	Count
272	C3,*Outside*	2
273	C1,C21,C31,*Outside*,*Young*	2
274	C34,C39,*Young*	2
275	C19,C4,*Young*	2
276	C26,*Outside*,*Seroconverted*,*Young*	2
277	C32,*Seroconverted*	2
278	C43,*Outside*,*Seroconverted*,*Young*	2
279	C25,C39,*Outside*,*Young*	2
280	C21,C24,*Outside*,*Young*	2
281	C23,C38,*Outside*,*Young*	2
282	C13,*Seroconverted*	2
283	C20,C35,*Outside*,*Young*	2
284	C23,C37,*Outside*,*Young*	2
285	C44,*Outside*,*Seroconverted*,*Young*	2
286	C21,C35,*Outside*,*Young*	2
287	C18,*Outside*	2
288	C29,C43,*Outside*,*Young*	2
289	C1,C13,*Outside*,*Young*	2
290	C38,C42,*Young*	2
291	C31,C34,*Young*	2
292	C18,C4,*Young*	2
293	C31,C34,*Outside*,*Young*	2
294	C27,C42,*Young*	2
295	C39,*Seroconverted*	2
296	C23,C8,*Young*	2
297	C6,*Seroconverted*	2
298	C35,C6,*Young*	2
299	C21,C36,*Young*	2
300	C1,C31,*Outside*,*Young*	2
301	C33,*Outside*,*Seroconverted*,*Young*	2
302	C2,C32,*Young*	2
303	C23,C37,*Young*	2
304	C18,*Outside*,*Seroconverted*,*Young*	2
305	C24,C36,*Young*	2
306	C1,*Seroconverted*,*Young*	2
307	C16,C40,*Outside*,*Young*	1
308	C12,C42,*Young*	1
309	C10,C35,*Young*	1
310	C41,C43	1
311	C23,C7,*Young*	1
312	C25,C37,*Seroconverted*,*Young*	1
313	C12,C27,*Young*	1
314	C19,C33,*Outside*,*Young*	1
315	C16,C33,*Outside*,*Young*	1
316	C10,C19,*Outside*,*Seroconverted*,*Young*	1
317	C24,C38,*Young*	1
318	C20,C33,*Young*	1
319	C11,C7,*Young*	1
320	C28,*Seroconverted*	1
321	C1,C34,*Outside*,*Seroconverted*,*Young*	1
322	C10,C11,*Seroconverted*,*Young*	1
323	C41,*Outside*,*Seroconverted*,*Young*	1
324	C11,*Seroconverted*	1
325	C16,C39,*Young*	1
326	C16,C39,*Outside*,*Young*	1
327	C8,*Seroconverted*	1
328	C25,C8,*Outside*,*Young*	1
329	C29,*Outside*,*Seroconverted*	1
330	C27,C8,*Outside*,*Young*	1
331	C19,C34,*Outside*	1
332	C4,*Outside*,*Seroconverted*	1
333	C23,C8,*Outside*,*Young*	1
334	C20,C31,*Outside*,*Young*	1
335	C1,C3,*Young*	1
336	C39,C40,*Seroconverted*,*Young*	1
337	C3,C34	1
338	C16,C34,*Outside*,*Seroconverted*	1

Table E – continued from previous page

Id.	Variables with level “yes”	Count
339	C15,C42	1
340	C11,C22,*Young*	1
341	C20,C36,*Outside*,*Young*	1
342	C24,C39,*Young*	1
343	C41,*Seroconverted*	1
344	C2,C31,*Seroconverted*,*Young*	1
345	C16,C3,*Young*	1
346	C36,C8,*Young*	1
347	C31,C36,*Young*	1
348	C1,C17,C34,*Young*	1
349	C5,*Outside*	1
350	C40,C9	1
351	C20,C31,*Seroconverted*,*Young*	1
352	C13,C41,*Outside*,*Seroconverted*,*Young*	1
353	C15,C27,*Young*	1
354	C21,C7,*Outside*,*Young*	1
355	C19,*Outside*,*Seroconverted*,*Young*	1
356	C14,C28,*Young*	1
357	C15,C42,*Outside*,*Young*	1
358	C12,C27	1
359	C14,*Outside*,*Seroconverted*	1
360	C14,*Seroconverted*	1
361	C11,C9,*Outside*,*Young*	1
362	C21,C9,*Young*	1
363	C42,C8,*Young*	1
364	C10,C11,*Outside*,*Young*	1
365	C28,C34,*Outside*,*Seroconverted*,*Young*	1
366	C13,C4,*Young*	1
367	C45,*Seroconverted*	1
368	C15,*Seroconverted*	1
369	C16,C19,C31,*Outside*,*Young*	1
370	C37,C8,*Young*	1
371	C36,C6,*Outside*,*Young*	1
372	C24,*Seroconverted*	1
373	C3,C32,*Young*	1
374	C18,C6,*Young*	1
375	C14,C16,*Young*	1
376	C23,C32,*Young*	1
377	C13,C34,*Outside*	1
378	C7,C9,*Young*	1
379	C37,C7,*Young*	1
380	C37,C7,*Outside*,*Young*	1
381	C13,C43,*Seroconverted*,*Young*	1
382	C19,C9,*Outside*,*Young*	1
383	C16,C17,C6,*Outside*,*Young*	1
384	C11,C24,*Outside*,*Young*	1
385	C27,*Seroconverted*	1
386	C2,C20,*Outside*,*Young*	1
387	C4,C8,*Outside*,*Seroconverted*,*Young*	1
388	C20,C6	1
389	C18,*Seroconverted*	1
390	C18,C31,*Outside*,*Young*	1
391	C11,C41	1
392	C11,C41,*Young*	1
393	C29,C44,*Young*	1
394	C20,C5,*Outside*,*Young*	1
395	C28,C43,*Young*	1
396	C2,C34,C9,*Young*	1
397	C25,C34,*Outside*,*Young*	1
398	C14,C24,*Young*	1
399	C11,C41,*Seroconverted*,*Young*	1
400	C19,C22,C3,*Outside*,*Young*	1
401	C27,*Outside*,*Seroconverted*	1
402	C23,C8,*Outside*,*Seroconverted*,*Young*	1
403	C32,*Outside*,*Seroconverted*	1
404	C15,C38,*Young*	1
405	C32,*Outside*	1
406	C22,C37,*Outside*	1

Table E – continued from previous page

Id.	Variables with level "yes"	Count
407	C31,C38,*Outside*,*Seroconverted*,*Young*	1
408	C37,C38,*Outside*,*Young*	1
409	C8,C9,*Outside*	1
410	C19,C41,*Outside*,*Young*	1
411	C3,C38,*Young*	1
412	C38,C7,*Outside*,*Young*	1
413	C14,C32,*Young*	1
414	C45,C6,*Young*	1
415	C13,C5,*Outside*,*Young*	1
416	C4,*Seroconverted*	1
417	C6,*Outside*,*Seroconverted*	1
418	C18,C34,*Outside*,*Young*	1
419	C28,C39	1
420	C17,C18,*Young*	1
421	C3,C33,*Seroconverted*,*Young*	1
422	C34,C35,*Young*	1
423	C15,C31,*Young*	1
424	C24,C36,*Outside*,*Seroconverted*,*Young*	1
425	C21,C34,*Seroconverted*,*Young*	1
426	C12,C21,*Young*	1
427	C33,C43,*Young*	1
428	C16,C43,*Young*	1
429	C34,C43,*Young*	1
430	C12,C9,*Outside*,*Young*	1
431	C3,*Seroconverted*	1
432	C23,C38,*Outside*	1
433	C16,C34,*Seroconverted*,*Young*	1
434	C33,*Outside*	1
435	C12,C29,*Young*	1
436	C35,*Outside*,*Seroconverted*	1
437	C20,C35	1
438	C10,C27,*Outside*,*Young*	1
439	C14,C20,*Outside*,*Young*	1
440	C20,C35,*Young*	1
441	C29,C43,*Young*	1
442	C23,C36,*Seroconverted*,*Young*	1
443	C19,C30,*Young*	1
444	C13,C30,*Young*	1
445	C10,C12,*Young*	1
446	C29,C4,*Outside*,*Young*	1
447	C12,*Seroconverted*	1
448	C10,C32,*Young*	1
449	C12,C31,*Outside*,*Young*	1
450	C12,C15,*Outside*,*Seroconverted*,*Young*	1
451	C24,C37,*Young*	1
452	C25,C40,*Young*	1
453	C15,C3,*Outside*,*Young*	1
454	C23,C33,*Young*	1
455	C10,C11,*Young*	1
456	C34,C9,*Young*	1
457	C2,C31,*Outside*	1
458	C12,C14,*Young*	1
459	C15,*Outside*,*Seroconverted*	1
460	C15,C17,*Outside*,*Seroconverted*,*Young*	1
461	C15,C17,*Young*	1
462	C19,C3,*Young*	1
463	C14,C5,*Outside*	1
464	C17,C32,*Outside*,*Young*	1
465	C22,C38,*Seroconverted*,*Young*	1
466	C44,*Outside*,*Seroconverted*	1
467	C34,C40,*Outside*,*Young*	1
468	C19,C33,*Young*	1
469	C42,C8	1
470	C45,*Seroconverted*,*Young*	1
471	C19,C23,*Outside*	1
472	C2,*Seroconverted*	1
473	C21,C36,*Outside*,*Young*	1
474	C26,*Seroconverted*,*Young*	1

Table E – continued from previous page

Id.	Variables with level "yes"	Count
475	C14,C16,*Outside*,*Seroconverted*,*Young*	1
476	C1,C19,*Outside*,*Young*	1
477	C39,C7,*Young*	1
478	C34,C36,*Young*	1
479	C30,C9,*Outside*,*Young*	1
480	C15,C8,*Outside*,*Young*	1
481	C23,C43,*Young*	1
482	C21,C34,*Outside*,*Young*	1
483	C1,C6,*Outside*,*Young*	1
484	C12,C19,*Outside*,*Young*	1
485	C41,*Outside*,*Seroconverted*	1
486	C15,C30,*Young*	1
487	C30,*Outside*,*Seroconverted*,*Young*	1
488	C20,C39,*Outside*,*Young*	1
489	C18,C35,*Young*	1
490	C23,C9,*Outside*,*Young*	1
491	C19,C34,*Outside*,*Young*	1
492	C18,C23,*Outside*,*Young*	1
493	C18,C20,*Outside*,*Seroconverted*,*Young*	1
494	C1,C34,*Outside*	1
495	C20,C40,*Outside*	1
496	C26,C41,*Outside*,*Young*	1
497	C10,C23,*Young*	1
498	C10,C23,*Outside*,*Young*	1
499	C33,C34,*Young*	1
500	C2,*Outside*	1
501	C20,C34,*Young*	1
502	C36,C37,*Young*	1
503	C20,*Seroconverted*	1
504	C19,C24,*Young*	1
505	C11,C12,*Outside*,*Young*	1
506	C16,C5,*Outside*,*Young*	1
507	C32,C37,*Seroconverted*,*Young*	1
508	C25,C45,*Young*	1
509	C34,C36	1
510	C24,C7,*Young*	1
511	C19,C40,*Young*	1
512	C28,C40,*Young*	1
513	C27,C38	1
514	C1,C3,*Outside*,*Young*	1
515	C1,C25,*Outside*,*Young*	1
516	C12,C42,*Outside*,*Young*	1
517	C16,C34,*Outside*,*Seroconverted*,*Young*	1
518	C36,C39,*Seroconverted*,*Young*	1
519	C35,C5,*Young*	1
520	C16,C4,C5,*Young*	1
521	C3,C34,*Outside*,*Young*	1
522	C2,C32,*Outside*,*Young*	1
523	C2,C34,*Outside*,*Young*	1
524	C7,*Outside*,*Seroconverted*	1
525	C5,C7,*Young*	1
526	C27,C42,*Outside*,*Young*	1
527	C33,*Outside*,*Seroconverted*	1
528	C14,C43,*Outside*,*Seroconverted*,*Young*	1
529	C19,C8,*Young*	1
530	C38,C9,*Young*	1
531	C16,C2,*Young*	1
532	C42,*Outside*,*Seroconverted*	1
533	C27,C3,*Outside*,*Young*	1
534	C31,C33,*Outside*	1
535	C17,C3,C31,*Outside*,*Young*	1
536	C16,C3,*Seroconverted*,*Young*	1
537	C18,C40,*Young*	1
538	C31,C4,*Young*	1
539	C14,C17,*Young*	1
540	C43,C7,*Young*	1
541	C27,C30	1
542	C2,C31,*Young*	1

Table E – continued from previous page

Id.	Variables with level “yes”	Count
543	C17,C26,*Seroconverted*,*Young*	1
544	C17,C32,*Seroconverted*	1
545	C29,C30,*Outside*,*Young*	1
546	C20,C31,C5,*Young*	1
547	C16,C17,*Outside*,*Young*	1
548	C23,C9,*Young*	1
549	C23,C37,*Outside*	1
550	C34,C7,*Young*	1
551	C21,C8,*Outside*,*Young*	1
552	C15,C31	1
553	C10,C33,*Outside*,*Young*	1
554	C27,C39,*Outside*,*Young*	1
555	C22,C7,*Outside*,*Young*	1
556	C27,C38,*Young*	1
557	C12,C15,*Young*	1
558	C12,C22,*Young*	1
559	C10,C42,*Outside*,*Young*	1
560	C10,C33,C42,*Outside*,*Young*	1
561	C44,C8,*Young*	1
562	C45,*Outside*	1
563	C3,C34,*Young*	1
564	C19,C21,*Young*	1
565	C3,C32,*Outside*,*Young*	1
566	C23,C7,*Outside*,*Young*	1
567	C34,C5,*Outside*,*Seroconverted*,*Young*	1
568	C32,C4,*Young*	1
569	C15,C35,*Outside*,*Young*	1
570	C2,C7,*Seroconverted*,*Young*	1
571	C20,*Outside*,*Seroconverted*	1
572	C30,C43,*Young*	1
573	C30,C34,*Young*	1
574	C22,C6,*Outside*,*Young*	1
575	C19,C20,*Outside*,*Young*	1
576	C13,C34,*Young*	1
577	C17,C27,*Seroconverted*,*Young*	1
578	C31	1
579	C1,C31,*Seroconverted*,*Young*	1
580	C1,C31	1
581	C3,C34,*Seroconverted*,*Young*	1
582	C1,C31,*Outside*	1
583	C1,C3,*Outside*,*Seroconverted*,*Young*	1
584	C31,*Outside*	1
585	C1,C16,*Outside*,*Young*	1
586	C1,*Outside*	1
587	C1,C34,*Outside*,*Young*	1
588	C19,C3,C5,*Outside*,*Young*	1
589	C15,C23,C37	1
590	C33,C34,*Outside*,*Young*	1
591	C38,C7,*Seroconverted*	1
592	C16,C34,*Outside*,*Young*	1
593	C1,*Outside*,*Seroconverted*,*Young*	1
594	C16,*Outside*,*Seroconverted*,*Young*	1
595	C17,*Outside*,*Seroconverted*,*Young*	1
596	C5,C9,*Outside*	1
597	C3,C5,*Outside*,*Young*	1
598	C2,C20,*Young*	1

	Young			
	No		Yes	
	Outside		Outside	
**Seroconverted**	No	Yes	No	Yes
No	2232	242	5379	1847
Yes	210	35	1559	654

Table B. Cross-classification of 12,158 women that participated in the study by their HIV seroconversion status (*Seroconverted*: Yes/No), whether they moved outside the study area (*Outside*: Yes/No) and whether they were less than 30 years old at the start of the study (*Young*: Yes/No).

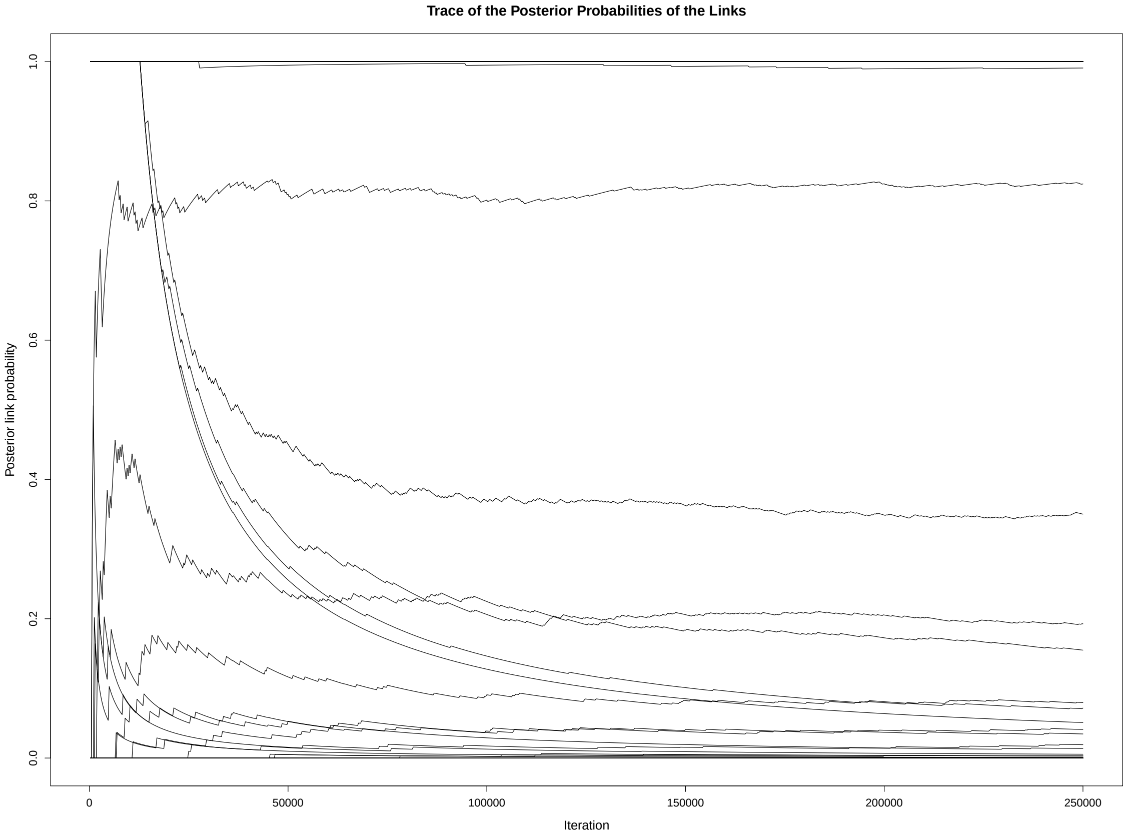

Fig. C. Convergence plot of the BDMCMC algorithm showing the estimated posterior inclusion probabilities of edges in graphs associated with men's mobility.

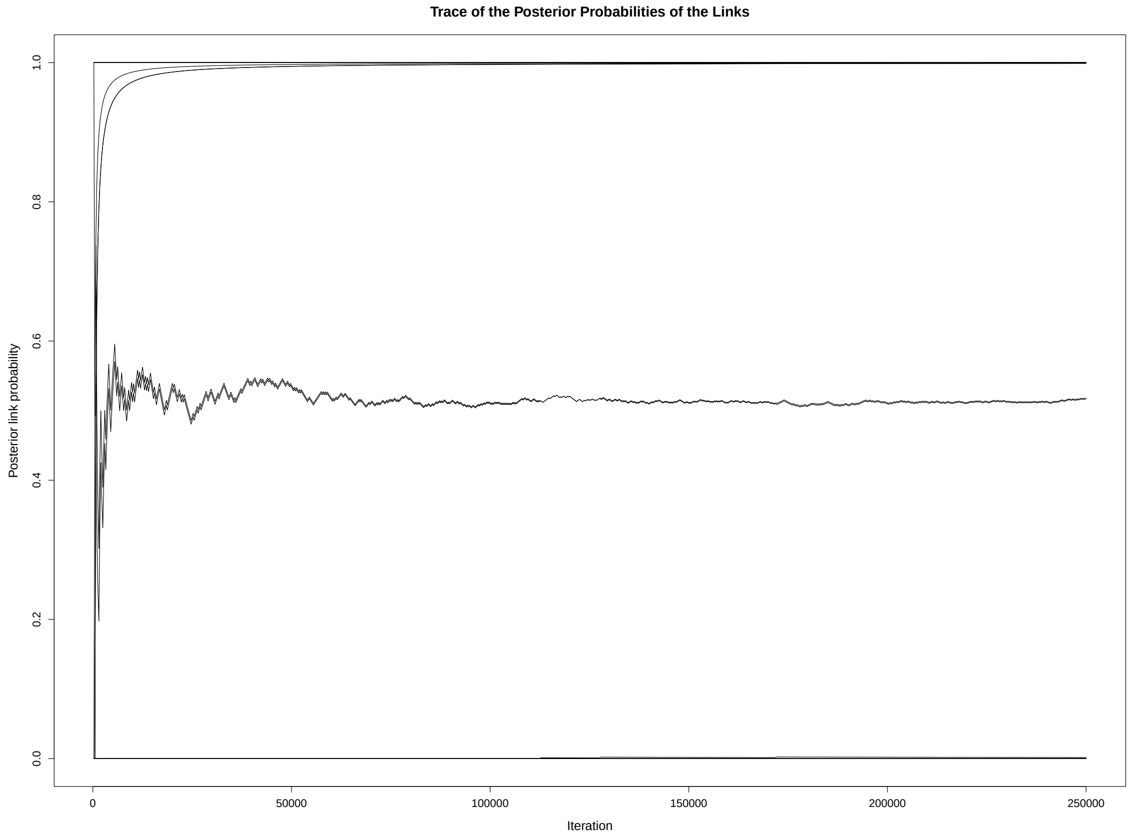

FIG. D. Convergence plot of the BDMCMC algorithm showing the estimated posterior inclusion probabilities of edges in graphs associated with women's mobility.

Table F: Cells with positive counts in the 48-dimensional dichotomous mobility table for women. Each row in the table is associated with one positive cell count. The first column gives the index of each count when the cells are ordered in decreasing order of their counts. The second column gives the names of the variables that take value "yes" for that count; the variables whose name do not appear take value "no". The third column gives the value of the cell count.

Id.	Variables with level "yes"	Count
1	C22, *Young*	185
2	C10, *Young*	176
3	C25, *Young*	175
4	C39, *Young*	172
5	C7, *Young*	171
6	C40, *Young*	166
7	C37, *Young*	163
8	C41, *Young*	152
9	C11, *Young*	146

Table F – continued from previous page

Id.	Variables with level "yes"	Count
10	C24, *Young*	143
11	C20, *Young*	142
12	C36, *Young*	141
13	*Outside*, *Young*	140
14	C13, *Young*	135
15	C34, *Young*	129
16	C38, *Young*	128
17	C27, *Young*	127
18	C15, *Young*	125
19	C14, *Young*	125
20	C44, *Young*	125
21	C45, *Young*	124
22	C21, *Young*	123
23	C43, *Young*	123
24	C42, *Young*	119
25	C23, *Young*	114
26	C29, *Young*	109
27	C17, *Young*	104
28	C30, *Young*	99
29	C5, *Young*	99
30	C6, *Young*	98
31	C4, *Young*	97
32	C8, *Young*	97
33	C26, *Young*	96
34	C3, *Young*	96
35	C35, *Young*	95
36	C9, *Young*	95
37	C19, *Young*	90
38	C22	90
39	C12, *Young*	87
40	C28, *Young*	82
41	C2, *Young*	78
42	C40	75
43	C25	75
44	C32, *Young*	74
45	C10	70
46	C39	70
47	C38	67
48	C24	64
49	C37	62
50	C13	61
51	C41	59
52	C8	59
53	C7	59
54	C15	59
55	C22, *Seroconverted*, *Young*	59
56	C14	59
57	C18, *Young*	58
58	C20	57
59	C10, *Seroconverted*, *Young*	55
60	C22, *Outside*, *Young*	55
61	C11	54
62	C21	54
63	C45	53
64	C33, *Young*	53
65	C43	51
66	C11, *Outside*, *Young*	51
67	C40, *Outside*, *Young*	51
68	C10, *Outside*, *Young*	51
69	C25, *Outside*, *Young*	51
70	C36, *Seroconverted*, *Young*	50
71	C41, *Outside*, *Young*	50
72	C44	50
73	C4	49
74	C39, *Outside*, *Young*	49
75	C26	49
76	C7, *Outside*, *Young*	48
77	C40, *Seroconverted*, *Young*	48

Table F – continued from previous page

Id.	Variables with level "yes"	Count
78	C29	48
79	C20, *Outside*, *Young*	48
80	C36	47
81	C37, *Outside*, *Young*	47
82	C38, *Outside*, *Young*	47
83	C27, *Seroconverted*, *Young*	47
84	C5, *Seroconverted*, *Young*	46
85	C23	46
86	C27, *Outside*, *Young*	46
87	C37, *Seroconverted*, *Young*	45
88	C27	45
89	C8, *Outside*, *Young*	45
90	C34	44
91	C5	44
92	C32	44
93	C24, *Outside*, *Young*	44
94	C30	43
95	C21, *Outside*, *Young*	43
96	C7, *Seroconverted*, *Young*	43
97	C42	43
98	C15, *Outside*, *Young*	43
99	C39, *Seroconverted*, *Young*	43
100	C21, *Seroconverted*, *Young*	43
101	C23, *Outside*, *Young*	42
102	C20, *Seroconverted*, *Young*	42
103	C35	42
104	C25, *Seroconverted*, *Young*	42
105	C23, *Seroconverted*, *Young*	42
106	*Outside*, *Seroconverted*, *Young*	42
107	C3	42
108	C43, *Outside*, *Young*	41
109	C19	40
110	C26, *Outside*, *Young*	40
111	C14, *Outside*, *Young*	40
112	C14, *Seroconverted*, *Young*	39
113	C35, *Seroconverted*, *Young*	39
114	C16, *Young*	39
115	C34, *Seroconverted*, *Young*	38
116	C13, *Outside*, *Young*	38
117	C42, *Outside*, *Young*	38
118	C4, *Seroconverted*, *Young*	37
119	*Outside*	37
120	C12	36
121	C28	36
122	C15, *Seroconverted*, *Young*	36
123	C41, *Seroconverted*, *Young*	36
124	C11, *Seroconverted*, *Young*	35
125	C29, *Outside*, *Young*	35
126	C6	35
127	C13, *Seroconverted*, *Young*	35
128	C18	35
129	C17	34
130	C26, *Seroconverted*, *Young*	33
131	C36, *Outside*, *Young*	33
132	C38, *Seroconverted*, *Young*	33
133	C44, *Outside*, *Young*	33
134	C30, *Outside*, *Young*	32
135	C45, *Outside*, *Young*	32
136	C35, *Outside*, *Young*	32
137	C8, *Seroconverted*, *Young*	32
138	C2, *Seroconverted*, *Young*	32
139	C24, *Seroconverted*, *Young*	31
140	C9, *Outside*, *Young*	31
141	C17, *Seroconverted*, *Young*	31
142	C12, *Seroconverted*, *Young*	31
143	C44, *Seroconverted*, *Young*	30
144	C42, *Seroconverted*, *Young*	30
145	C19, *Outside*, *Young*	30

Table F – continued from previous page

Id.	Variables with level “yes”	Count
146	C6,*Seroconverted*,*Young*	29
147	C29,*Seroconverted*,*Young*	28
148	C34,*Outside*,*Young*	27
149	C45,*Seroconverted*,*Young*	27
150	C2	27
151	C1,*Young*	27
152	C5,*Outside*,*Young*	25
153	C43,*Seroconverted*,*Young*	25
154	C12,*Outside*,*Young*	25
155	C31,*Young*	25
156	C30,*Seroconverted*,*Young*	24
157	C32,*Seroconverted*,*Young*	24
158	C21,*Outside*,*Seroconverted*,*Young*	24
159	C4,*Outside*,*Young*	24
160	C10,*Outside*,*Seroconverted*,*Young*	23
161	C36,*Outside*,*Seroconverted*,*Young*	23
162	C3,*Seroconverted*,*Young*	23
163	C9	22
164	C9,*Seroconverted*,*Young*	22
165	C28,*Seroconverted*,*Young*	21
166	C13,*Outside*,*Seroconverted*,*Young*	21
167	C17,*Outside*,*Young*	21
168	C33	20
169	C39,*Outside*,*Seroconverted*,*Young*	20
170	C38,*Outside*,*Seroconverted*,*Young*	20
171	C28,*Outside*,*Young*	19
172	C27,*Outside*,*Seroconverted*,*Young*	19
173	C33,*Outside*,*Young*	19
174	C35,*Outside*,*Seroconverted*,*Young*	19
175	C18,*Outside*,*Young*	19
176	C22,*Outside*,*Seroconverted*,*Young*	18
177	C40,*Outside*,*Seroconverted*,*Young*	18
178	C32,*Outside*,*Young*	18
179	C23,*Outside*,*Seroconverted*,*Young*	17
180	C2,*Outside*,*Young*	17
181	C6,*Outside*,*Young*	16
182	C7,*Outside*,*Seroconverted*,*Young*	15
183	C3,*Outside*,*Young*	15
184	C42,*Outside*,*Seroconverted*,*Young*	15
185	C20,*Outside*,*Seroconverted*,*Young*	15
186	C24,*Outside*,*Seroconverted*,*Young*	14
187	C11,*Outside*,*Seroconverted*,*Young*	14
188	C32,*Outside*,*Seroconverted*,*Young*	14
189	C12,*Outside*,*Seroconverted*,*Young*	14
190	C26,C41,*Young*	13
191	C43,*Outside*,*Seroconverted*,*Young*	13
192	C15,*Outside*,*Seroconverted*,*Young*	13
193	C29,*Outside*,*Seroconverted*,*Young*	13
194	C37,*Outside*,*Seroconverted*,*Young*	13
195	C19,*Seroconverted*,*Young*	13
196	C2,*Outside*,*Seroconverted*,*Young*	13
197	C16	12
198	C25,*Outside*,*Seroconverted*,*Young*	12
199	C14,*Outside*,*Seroconverted*,*Young*	12
200	C17,*Outside*,*Seroconverted*,*Young*	12
201	C23,*Outside*	11
202	C19,*Outside*,*Seroconverted*,*Young*	11
203	C28,*Outside*,*Seroconverted*,*Young*	10
204	C26,*Outside*,*Seroconverted*,*Young*	10
205	C45,*Outside*,*Seroconverted*,*Young*	10
206	C29,*Outside*	9
207	C37,*Outside*	9
208	C1,*Outside*,*Young*	9
209	C8,*Outside*,*Seroconverted*,*Young*	9
210	C39,*Seroconverted*	9
211	C31	9
212	C10,*Outside*	8

Table F – continued from previous page

Id.	Variables with level "yes"	Count
213	C27,*Seroconverted*	8
214	C23,*Seroconverted*	8
215	C42,*Seroconverted*	8
216	C18,*Outside*,*Seroconverted*,*Young*	8
217	C44,*Seroconverted*	8
218	C14,*Seroconverted*	8
219	C34,*Outside*,*Seroconverted*,*Young*	8
220	C16,*Seroconverted*,*Young*	8
221	C13,*Outside*	8
222	C1,*Seroconverted*,*Young*	8
223	C31,*Seroconverted*,*Young*	8
224	C6,*Seroconverted*	7
225	C33,*Seroconverted*,*Young*	7
226	C25,*Seroconverted*	7
227	C18,*Seroconverted*,*Young*	7
228	C21,*Seroconverted*	7
229	*Outside*,*Seroconverted*	7
230	C3,*Seroconverted*	7
231	C13,*Seroconverted*	7
232	C44,*Outside*,*Seroconverted*,*Young*	7
233	C40,*Outside*	7
234	C36,*Outside*	7
235	C6,*Outside*,*Seroconverted*,*Young*	7
236	C38,*Seroconverted*	7
237	C30,*Outside*,*Seroconverted*,*Young*	6
238	C22,*Seroconverted*	6
239	C7,*Seroconverted*	6
240	C15,*Seroconverted*	6
241	C3,*Outside*,*Seroconverted*,*Young*	6
242	C5,*Seroconverted*	6
243	C24,*Outside*	6
244	C34,*Seroconverted*	6
245	C22,*Outside*	6
246	C35,*Seroconverted*	6
247	C10,*Seroconverted*	6
248	C4,*Outside*,*Seroconverted*,*Young*	5
249	C25,*Outside*	5
250	C20,*Seroconverted*	5
251	C11,C41,*Young*	5
252	C10,C11,*Outside*,*Young*	5
253	C41,*Outside*	5
254	C45,*Outside*	5
255	C41,*Outside*,*Seroconverted*,*Young*	5
256	C27,*Outside*	5
257	C11,*Outside*	5
258	C9,*Outside*,*Seroconverted*,*Young*	5
259	C12,*Outside*	5
260	C35,*Outside*	5
261	C7,*Outside*	5
262	C1	5
263	C30,*Seroconverted*	4
264	C11,C41,*Outside*,*Young*	4
265	C29,C43,*Outside*,*Young*	4
266	C13,C43,*Young*	4
267	C19,*Seroconverted*	4
268	C17,C2,*Young*	4
269	C26,*Outside*	4
270	C9,*Outside*	4
271	C11,*Seroconverted*	4
272	C24,*Seroconverted*	4
273	C28,*Seroconverted*	4
274	C8,*Seroconverted*	4
275	C38,*Outside*	4
276	C31,*Outside*,*Seroconverted*,*Young*	4
277	C1,C31,*Young*	4
278	C34,C35,*Young*	3
279	C26,C41,*Outside*,*Seroconverted*,*Young*	3

Table F – continued from previous page

Id.	Variables with level "yes"	Count
280	C2,*Seroconverted*	3
281	C39,*Outside*	3
282	C14,C29,*Young*	3
283	C17,*Seroconverted*	3
284	C41,*Seroconverted*	3
285	C8,*Outside*	3
286	C44,C45,*Young*	3
287	C5,*Outside*,*Seroconverted*,*Young*	3
288	C27,C42,*Young*	3
289	C45,*Outside*,*Seroconverted*	3
290	C33,*Seroconverted*	3
291	C12,C13,*Young*	3
292	C5,*Outside*	3
293	C20,*Outside*,*Seroconverted*	3
294	C34,*Outside*	3
295	C38,C7,*Young*	3
296	C42,*Outside*	3
297	C29,C44,*Young*	3
298	C12,*Seroconverted*	3
299	C43,*Seroconverted*	3
300	C20,C5,*Outside*,*Seroconverted*,*Young*	3
301	C32,*Seroconverted*	3
302	C37,*Seroconverted*	3
303	C32,*Outside*	3
304	C19,*Outside*	3
305	C17,*Outside*	3
306	C26,*Seroconverted*	3
307	C1,C16,*Young*	3
308	C11,C12	2
309	C10,C9,*Young*	2
310	C23,C32,*Outside*,*Young*	2
311	C25,C39,*Young*	2
312	C19,C25,*Outside*,*Young*	2
313	C20,C35,*Outside*,*Young*	2
314	C13,C42,*Outside*,*Young*	2
315	C37,C38,*Outside*,*Young*	2
316	C29,C45	2
317	C13,C9,*Outside*,*Young*	2
318	C4,*Seroconverted*	2
319	C19,C43,*Young*	2
320	C2,C31,*Outside*	2
321	C34,C39,*Young*	2
322	C10,C24,*Young*	2
323	C23,C8,*Young*	2
324	C36,C37,*Seroconverted*,*Young*	2
325	C14,C28	2
326	C20,*Outside*	2
327	C37,C7,*Young*	2
328	C12,C42,*Young*	2
329	C15,C19,*Outside*,*Young*	2
330	C37,C7	2
331	C15,*Outside*	2
332	C35,C6	2
333	C22,C9,*Seroconverted*,*Young*	2
334	C20,C5,*Seroconverted*,*Young*	2
335	C20,C7,*Young*	2
336	C29,C42,*Outside*,*Young*	2
337	C34,C45,*Outside*,*Young*	2
338	C36,C6,*Seroconverted*,*Young*	2
339	C21,C6,*Young*	2
340	C26,C43,*Outside*,*Young*	2
341	C15,C9,*Young*	2
342	C18,C4,*Outside*,*Young*	2
343	C23,C37,*Outside*,*Young*	2
344	C6,C7,*Outside*,*Young*	2
345	C13,C43,*Seroconverted*,*Young*	2
346	C35,*Outside*,*Seroconverted*	2

Table F – continued from previous page

Id.	Variables with level "yes"	Count
347	C29,*Seroconverted*	2
348	C14,C27,*Young*	2
349	C14,C18,*Outside*,*Young*	2
350	C22,C38,*Young*	2
351	C43,*Outside*	2
352	C36,C42,*Young*	2
353	C31,*Outside*	2
354	C36,C7,*Outside*,*Young*	2
355	C44,C45,*Outside*,*Young*	2
356	C9,*Seroconverted*	2
357	C13,C43	2
358	C40,*Seroconverted*	2
359	C11,C41,*Seroconverted*,*Young*	2
360	C37,C8,*Young*	2
361	C31,*Outside*,*Young*	2
362	C26,C41,*Outside*,*Young*	2
363	C28,*Outside*	2
364	C12,C43,*Outside*,*Young*	2
365	C6,*Outside*	2
366	C14,C29,*Seroconverted*,*Young*	2
367	C15,C3,*Young*	2
368	C35,C6,*Young*	2
369	C29,C30,*Young*	2
370	C21,C7,*Young*	2
371	C31,C34,*Outside*,*Seroconverted*,*Young*	2
372	C1,C22,*Outside*,*Young*	2
373	C15,C17,*Outside*,*Young*	2
374	C19,C25,*Young*	2
375	C14,C26,*Young*	2
376	C25,C40	2
377	C39,C40	2
378	C45,*Seroconverted*	2
379	C10,C40	2
380	C2,C40,*Young*	2
381	C27,C5,*Outside*,*Young*	2
382	C6,*Outside*,*Seroconverted*	2
383	C38,C42,*Young*	2
384	C2,C5	2
385	C36,*Seroconverted*	2
386	C16,*Outside*,*Seroconverted*,*Young*	2
387	C13,C26,*Young*	2
388	C19,C4,*Young*	2
389	C10,C23,*Young*	2
390	C3,C36,*Young*	2
391	C22,C37,*Outside*,*Young*	2
392	C17,C2,*Seroconverted*,*Young*	2
393	C13,C27,*Young*	2
394	C24,C39,*Outside*,*Young*	2
395	C4,C5,*Young*	2
396	C27,C42	2
397	C14,*Outside*	2
398	C21,*Outside*	2
399	C1,C31,*Outside*,*Young*	2
400	C1,*Outside*	2
401	C36,C8,*Young*	2
402	C10,C11,*Seroconverted*,*Young*	2
403	C27,C42,*Seroconverted*,*Young*	2
404	C16,C31,*Young*	2
405	C16,*Seroconverted*	2
406	C12,C42	1
407	C30,C45,*Young*	1
408	C14,C43,*Young*	1
409	C10,C35,*Young*	1
410	C41,C43	1
411	C24,C5,*Seroconverted*,*Young*	1
412	C16,C27	1
413	C2,C7,*Outside*,*Young*	1
414	C1,C16,C27,*Outside*,*Young*	1

Table F – continued from previous page

Id.	Variables with level “yes”	Count
415	C11,C41,*Outside*,*Seroconverted*,*Young*	1
416	C22,C7,*Young*	1
417	C23,C36,*Outside*,*Young*	1
418	C34,C40,*Seroconverted*,*Young*	1
419	C7,C9,*Outside*,*Young*	1
420	C14,C29,*Outside*	1
421	C34,C7,*Outside*,*Seroconverted*,*Young*	1
422	C33,C38,*Outside*,*Young*	1
423	C23,*Outside*,*Seroconverted*	1
424	C21,C44,*Young*	1
425	C25,*Outside*,*Seroconverted*	1
426	C24,C39,*Outside*	1
427	C27,C35,*Outside*	1
428	C15,C27,*Seroconverted*,*Young*	1
429	C16,C26,C4,*Seroconverted*,*Young*	1
430	C17,C29,*Seroconverted*,*Young*	1
431	C13,C16,*Outside*,*Young*	1
432	C10,C15,*Outside*,*Young*	1
433	C16,C5,*Young*	1
434	C44,*Outside*	1
435	C11,C34,*Seroconverted*,*Young*	1
436	C11,C12,C9,*Seroconverted*,*Young*	1
437	C19,C3,*Outside*,*Young*	1
438	C24,C36,*Young*	1
439	C19,C40,*Outside*,*Young*	1
440	C18,C45,*Outside*,*Young*	1
441	C41,C43,*Young*	1
442	C18,C45	1
443	C3,C45,*Outside*,*Seroconverted*,*Young*	1
444	C1,C2,C39	1
445	C3,C9	1
446	C10,C11	1
447	C32,C35,*Seroconverted*,*Young*	1
448	C10,C41,*Outside*	1
449	C1,C31,C32,*Outside*,*Seroconverted*,*Young*	1
450	C26,C30,*Outside*,*Seroconverted*,*Young*	1
451	C10,C37,*Young*	1
452	C17,C31,C38,*Young*	1
453	C25,C33,*Outside*,*Young*	1
454	C25,C40,*Outside*,*Young*	1
455	C3,C33,*Outside*	1
456	C14,C28,*Outside*,*Seroconverted*,*Young*	1
457	C2,C23,*Young*	1
458	C2,C23,*Seroconverted*,*Young*	1
459	C10,C41,*Outside*,*Seroconverted*,*Young*	1
460	C1,C35,*Outside*,*Seroconverted*,*Young*	1
461	C10,C20,*Outside*,*Young*	1
462	C15,C27,*Seroconverted*	1
463	C19,C23,*Young*	1
464	C15,C22,*Outside*,*Young*	1
465	C41,C43,*Outside*,*Seroconverted*,*Young*	1
466	C17,C43,*Outside*	1
467	C40,C5,*Outside*,*Young*	1
468	C21,C24,*Outside*,*Seroconverted*,*Young*	1
469	C21,C24,*Young*	1
470	C35,C36,C37,*Outside*	1
471	C36,C40,*Seroconverted*,*Young*	1
472	C32,C42,*Young*	1
473	C15,C43	1
474	C18,C3,*Seroconverted*,*Young*	1
475	C16,C44,*Seroconverted*	1
476	C23,C6,*Seroconverted*,*Young*	1
477	C16,C44,*Outside*,*Seroconverted*,*Young*	1
478	C6,C8,*Outside*,*Young*	1
479	C17,C34	1
480	C15,C30,*Outside*,*Young*	1
481	C1,C16,C25,*Outside*,*Seroconverted*	1

Table F – continued from previous page

Id.	Variables with level "yes"	Count
482	C24,C33,*Outside*,*Young*	1
483	C10,C3,*Young*	1
484	C18,C29,*Outside*,*Young*	1
485	C15,C4,*Outside*,*Young*	1
486	C43,*Outside*,*Seroconverted*	1
487	C12,C14,*Outside*	1
488	C27,C29,*Young*	1
489	C16,*Outside*	1
490	C13,C33,*Seroconverted*,*Young*	1
491	C20,C5,*Young*	1
492	C13,C14,*Young*	1
493	C12,C21,C9,*Seroconverted*,*Young*	1
494	C29,C44,*Outside*,*Young*	1
495	C29,C44	1
496	C11,C18,*Seroconverted*,*Young*	1
497	C21,C9,*Young*	1
498	C10,C32,*Outside*,*Young*	1
499	C10,C29,*Young*	1
500	C15,C42,*Young*	1
501	C19,C31,*Outside*,*Seroconverted*,*Young*	1
502	C21,C36,*Outside*,*Young*	1
503	C17,C25,*Outside*,*Young*	1
504	C18,C8,*Outside*,*Young*	1
505	C38,C41	1
506	C15,C19,*Young*	1
507	C35,C36,*Outside*,*Young*	1
508	C14,C41,*Young*	1
509	C32,C33,C38,*Outside*,*Young*	1
510	C13,C15	1
511	C20,C22,*Seroconverted*,*Young*	1
512	C31,C4,*Outside*,*Seroconverted*,*Young*	1
513	C23,C34,*Outside*,*Young*	1
514	C6,C7,*Young*	1
515	C2,C34,*Young*	1
516	C11,C13	1
517	C35,C6,*Seroconverted*	1
518	C11,C26,*Young*	1
519	C16,C7,*Outside*,*Young*	1
520	C13,C30,*Young*	1
521	C3,C45,*Young*	1
522	C23,C32,*Outside*,*Seroconverted*,*Young*	1
523	C18,C34,*Young*	1
524	C18,C19,*Young*	1
525	C13,C43,*Outside*,*Seroconverted*,*Young*	1
526	C11,C39,*Seroconverted*,*Young*	1
527	C25,C4,*Outside*,*Young*	1
528	C14,C28,*Seroconverted*	1
529	C40,C9,*Young*	1
530	C16,C2,*Young*	1
531	C17,C20,*Seroconverted*,*Young*	1
532	C20,C6,*Young*	1
533	C22,C6,*Young*	1
534	C19,C34	1
535	C39,C40,*Outside*,*Young*	1
536	C41,C9,*Young*	1
537	C21,C7,*Seroconverted*	1
538	C44,C9,*Outside*,*Young*	1
539	C11,C41	1
540	C13,C37,*Outside*,*Young*	1
541	C43,C45,*Outside*,*Young*	1
542	C2,C9,*Outside*,*Seroconverted*,*Young*	1
543	C15,C31,*Outside*,*Young*	1
544	C35,C9,*Young*	1
545	C30,C6,*Young*	1
546	C44,C45	1
547	C22,C6,*Seroconverted*,*Young*	1
548	C12,C30,*Outside*,*Young*	1
549	C1,C17,C29,*Outside*,*Young*	1

Table F – continued from previous page

Id.	Variables with level "yes"	Count
550	C11,C41,*Outside*	1
551	C16,C20,*Young*	1
552	C17,C2,*Outside*,*Seroconverted*,*Young*	1
553	C19,C2,*Young*	1
554	C24,C3,*Outside*,*Young*	1
555	C10,C9,*Outside*,*Seroconverted*,*Young*	1
556	C30,*Outside*,*Seroconverted*	1
557	C36,C39	1
558	C19,C32,*Young*	1
559	C11,C31,*Outside*,*Seroconverted*,*Young*	1
560	C25,C34,*Young*	1
561	C10,C11,*Outside*	1
562	C22,C37	1
563	C18,*Seroconverted*	1
564	C13,C27,*Outside*,*Young*	1
565	C15,C19,C38,*Outside*,*Young*	1
566	C42,C9,*Outside*,*Young*	1
567	C10,C34,*Young*	1
568	C20,C24	1
569	C13,C44,*Outside*,*Young*	1
570	C11,C27,*Outside*,*Young*	1
571	C45,C7,*Outside*,*Young*	1
572	C1,C15,*Outside*,*Young*	1
573	C13,C9,*Outside*,*Seroconverted*,*Young*	1
574	C19,C2,*Outside*,*Young*	1
575	C2,*Outside*	1
576	C7,C8,*Outside*,*Young*	1
577	C23,C38,*Outside*,*Seroconverted*,*Young*	1
578	C10,C37,*Seroconverted*,*Young*	1
579	C22,C38,*Outside*,*Young*	1
580	C20,C35,*Young*	1
581	C34,C42,*Seroconverted*,*Young*	1
582	C21,C22,*Young*	1
583	C1,C5,C7,*Outside*,*Seroconverted*,*Young*	1
584	C14,C28,*Young*	1
585	C13,C29,*Young*	1
586	C38,C7	1
587	C14,C8,*Young*	1
588	C14,C26,*Seroconverted*,*Young*	1
589	C10,C40,*Outside*,*Young*	1
590	C27,C44,C7,*Outside*,*Young*	1
591	C44,C7,*Outside*,*Young*	1
592	C29,C44,*Seroconverted*,*Young*	1
593	C10,C40,*Outside*,*Seroconverted*,*Young*	1
594	C12,C27,*Young*	1
595	C28,C43,*Young*	1
596	C28,C30,*Young*	1
597	C12,C18,*Outside*	1
598	C12,C42,*Seroconverted*,*Young*	1
599	C22,C24	1
600	C17,C27,*Outside*,*Young*	1
601	C33,C9,*Outside*,*Young*	1
602	C1,C4,*Outside*	1
603	C1,C4,*Young*	1
604	C21,C29,*Young*	1
605	C15,C36,*Seroconverted*,*Young*	1
606	C13,C30,*Outside*,*Young*	1
607	C12,C21,*Seroconverted*,*Young*	1
608	C15,C19,*Outside*,*Seroconverted*,*Young*	1
609	C15,C18,*Young*	1
610	C12,C43,*Young*	1
611	C36,C37	1
612	C20,C35,C6,*Young*	1
613	C24,C8,*Outside*,*Young*	1
614	C42,C43	1
615	C25,C43,*Outside*,*Young*	1
616	C37,C39,*Seroconverted*,*Young*	1

Table F – continued from previous page

Id.	Variables with level “yes”	Count
617	C12,C20,*Outside*,*Seroconverted*,*Young*	1
618	C15,C42,*Outside*,*Seroconverted*,*Young*	1
619	C12,C13	1
620	C18,C36,*Outside*,*Young*	1
621	C22,C40,*Young*	1
622	C24,C42,*Seroconverted*,*Young*	1
623	C30,C43,*Outside*,*Seroconverted*,*Young*	1
624	C30,C43,*Seroconverted*,*Young*	1
625	C25,C40,*Young*	1
626	C17,C2,*Outside*	1
627	C29,C45,*Young*	1
628	C23,C38,*Young*	1
629	C17,C4,*Young*	1
630	C22,C38,*Outside*	1
631	C33,C34,*Seroconverted*,*Young*	1
632	C16,C33,*Outside*,*Young*	1
633	C13,C28,*Young*	1
634	C27,C34,*Outside*,*Seroconverted*,*Young*	1
635	C18,C4,*Seroconverted*,*Young*	1
636	C16,C17,*Outside*,*Young*	1
637	C14,C29,C35,*Outside*,*Young*	1
638	C10,C3,*Seroconverted*,*Young*	1
639	C3,C39	1
640	C22,C26	1
641	C12,C29	1
642	C12,C43,*Seroconverted*,*Young*	1
643	C37,C38,*Young*	1
644	C10,C6,*Outside*,*Young*	1
645	C44,C8	1
646	C13,C39,*Outside*,*Young*	1
647	C19,C44,*Outside*,*Young*	1
648	C34,C39	1
649	C1,C32,*Outside*,*Young*	1
650	C25,C40,*Seroconverted*,*Young*	1
651	C10,C27,*Outside*	1
652	C38,C42,*Outside*,*Young*	1
653	C33,*Outside*,*Seroconverted*	1
654	C13,*Outside*,*Seroconverted*	1
655	C1,C2,C32,*Outside*,*Seroconverted*,*Young*	1
656	C35,C37,*Young*	1
657	C25,C4,*Outside*	1
658	C25,C4,*Young*	1
659	C13,C14,C37,*Young*	1
660	C23,C9,*Young*	1
661	C35,C40,*Young*	1
662	C10,C31,*Seroconverted*,*Young*	1
663	C25,C3,*Outside*,*Young*	1
664	C19,C34,*Young*	1
665	C10,C34,C40,*Young*	1
666	C26,C27,*Seroconverted*,*Young*	1
667	C26,C27,C33,*Outside*,*Young*	1
668	C26,C27,*Young*	1
669	C34,C37,*Outside*,*Young*	1
670	C23,C36,*Young*	1
671	C13,C39,*Young*	1
672	C20,C36,*Young*	1
673	C32,C39,*Outside*,*Seroconverted*,*Young*	1
674	C22,C5,*Seroconverted*,*Young*	1
675	C13,C30	1
676	C15,C3	1
677	C18,C36,*Young*	1
678	C29,C33,*Seroconverted*,*Young*	1
679	C10,C11,*Young*	1
680	C10,C21,*Outside*,*Young*	1
681	C16,C7,*Seroconverted*,*Young*	1
682	C22,C25	1
683	C22,C25,*Young*	1
684	C22,C25,*Outside*,*Young*	1

Table F – continued from previous page

Id.	Variables with level "yes"	Count
685	C12,C41,*Young*	1
686	C14,C27,*Seroconverted*,*Young*	1
687	C14,C19,*Outside*,*Seroconverted*,*Young*	1
688	C30,C41,*Outside*,*Young*	1
689	C2,C7,*Young*	1
690	C20,C4,*Seroconverted*,*Young*	1
691	C36,*Outside*,*Seroconverted*	1
692	C23,C37,C8,*Outside*,*Young*	1
693	C17,C5,*Outside*,*Young*	1
694	C15,C27,*Outside*,*Young*	1
695	C12,C36,*Young*	1
696	C14,C43	1
697	C14,C43,*Outside*,*Seroconverted*,*Young*	1
698	C1,C32,*Outside*	1
699	C15,C27,*Young*	1
700	C15,C9,*Outside*,*Seroconverted*,*Young*	1
701	C17,C24,*Outside*,*Young*	1
702	C23,C4,*Seroconverted*,*Young*	1
703	C14,C44,*Young*	1
704	C12,C17,*Outside*,*Seroconverted*,*Young*	1
705	C37,C40,*Seroconverted*,*Young*	1
706	C17,C27,*Seroconverted*,*Young*	1
707	C38,C8	1
708	C19,C20,C34,*Young*	1
709	C12,C24	1
710	C14,C15,*Young*	1
711	C41,C9	1
712	C19,C40,*Outside*	1
713	C30,C42,*Young*	1
714	C12,C15,C21,*Outside*,*Seroconverted*,*Young*	1
715	C23,C8,*Seroconverted*,*Young*	1
716	C30,C42,*Outside*,*Young*	1
717	C12,C22,*Outside*	1
718	C41,C9,*Seroconverted*,*Young*	1
719	C11,C8	1
720	C1,C42,C8	1
721	C14,C37,*Outside*,*Young*	1
722	C12,C8,*Outside*,*Young*	1
723	C37,C7,*Outside*,*Young*	1
724	C3,C35,*Seroconverted*,*Young*	1
725	C25,C38,*Outside*,*Seroconverted*,*Young*	1
726	C25,C38,*Seroconverted*,*Young*	1
727	C13,C45,*Young*	1
728	C12,C45,*Young*	1
729	C38,C4,*Young*	1
730	C1,C19,C21,*Outside*,*Seroconverted*,*Young*	1
731	C23,C25,*Seroconverted*,*Young*	1
732	C13,C24	1
733	C34,C39,C5,*Young*	1
734	C33,C37,C5,*Outside*,*Young*	1
735	C24,C37,*Outside*,*Young*	1
736	C16,C34,*Outside*	1
737	C17,C21,*Young*	1
738	C10,C38,C41,C8,*Young*	1
739	C19,C21,*Outside*,*Young*	1
740	C24,C40	1
741	C20,C39	1
742	C20,C8	1
743	C42,C9,*Seroconverted*,*Young*	1
744	C13,C15,*Young*	1
745	C14,C5	1
746	C27,C36,*Young*	1
747	C27,C3,*Outside*,*Young*	1
748	C19,C25,C34,*Outside*	1
749	C12,C41	1
750	C1,C2,*Outside*,*Seroconverted*,*Young*	1
751	C1,C2,*Young*	1
752	C26,C39,*Outside*,*Young*	1

Table F – continued from previous page

Id.	Variables with level "yes"	Count
753	C34,C5,*Young*	1
754	C34,C5,*Seroconverted*,*Young*	1
755	C25,C32,*Outside*	1
756	C23,C37	1
757	C12,C14,*Outside*,*Seroconverted*,*Young*	1
758	C15,C30	1
759	C18,C35,*Young*	1
760	C23,C41,C9,*Outside*,*Young*	1
761	C19,C31,*Outside*	1
762	C19,C31,*Outside*,*Young*	1
763	C23,C8	1
764	C21,C4,*Seroconverted*,*Young*	1
765	C27,C9,*Young*	1
766	C21,C35	1
767	C20,C33,*Outside*,*Young*	1
768	C24,C33,*Young*	1
769	C13,C28,*Seroconverted*,*Young*	1
770	C25,C34,*Seroconverted*,*Young*	1
771	C1,C34,*Outside*,*Seroconverted*	1
772	C11,*Outside*,*Seroconverted*	1
773	C1,C34,*Outside*,*Young*	1
774	C22,C39,*Young*	1
775	C10,C18,C3,*Young*	1
776	C24,C40,*Seroconverted*	1
777	C15,C38,*Outside*	1
778	C14,C15,C44,*Young*	1
779	C11,C40,*Young*	1
780	C19,C20,*Outside*,*Seroconverted*,*Young*	1
781	C2,C5,*Young*	1
782	C11,C12,*Outside*,*Young*	1
783	C11,C12,*Outside*,*Seroconverted*,*Young*	1
784	C23,C39,*Seroconverted*,*Young*	1
785	C1,C3,*Outside*,*Young*	1
786	C38,C8,*Young*	1
787	C5,C6,*Young*	1
788	C23,C35,C37,*Outside*,*Seroconverted*,*Young*	1
789	C39,C9,*Young*	1
790	C25,C34,C40,*Outside*,*Young*	1
791	C19,C20,*Outside*,*Young*	1
792	C34,C36	1
793	C34,C36,*Young*	1
794	C10,C12,*Young*	1
795	C17,C23,*Seroconverted*,*Young*	1
796	C14,C19,*Seroconverted*,*Young*	1
797	C24,*Outside*,*Seroconverted*	1
798	C14,C43,*Seroconverted*,*Young*	1
799	C11,C17,*Young*	1
800	C20,C33,*Young*	1
801	C39,C4,*Young*	1
802	C35,C39	1
803	C33,*Outside*,*Seroconverted*,*Young*	1
804	C22,C42,*Outside*,*Seroconverted*,*Young*	1
805	C11,C8,*Outside*,*Seroconverted*,*Young*	1
806	C13,C27	1
807	C36,C39,*Seroconverted*,*Young*	1
808	C19,C2,C35,*Young*	1
809	C19,C35,*Young*	1
810	C19,C33,*Outside*,*Young*	1
811	C3,C34	1
812	C3,C34,*Outside*,*Seroconverted*,*Young*	1
813	C44,C9,*Young*	1
814	C8,C9,*Outside*	1
815	C8,*Outside*,*Seroconverted*	1
816	C2,C32,*Outside*,*Young*	1
817	C32,C5,*Young*	1
818	C24,C8,*Young*	1
819	C27,*Outside*,*Seroconverted*	1
820	C35,C4,*Seroconverted*,*Young*	1

Table F – continued from previous page

Id.	Variables with level "yes"	Count
821	C12,C7,*Young*	1
822	C17,C29,C4,*Outside*,*Young*	1
823	C2,C30,*Outside*,*Young*	1
824	C23,C24,*Young*	1
825	C22,C32,*Outside*,*Seroconverted*,*Young*	1
826	C34,C41,*Seroconverted*,*Young*	1
827	C27,C39,*Outside*	1
828	C3,C32	1
829	C4,*Outside*	1
830	C17,*Outside*,*Seroconverted*	1
831	C2,C6,*Young*	1
832	C27,C40,*Outside*	1
833	C3,C31,*Outside*	1
834	C39,C5,*Seroconverted*,*Young*	1
835	C25,C39,C5,*Outside*	1
836	C1,C6,*Outside*,*Young*	1
837	C4,C41,*Outside*,*Young*	1
838	C21,C39,*Outside*,*Seroconverted*,*Young*	1
839	C26,C28,*Seroconverted*,*Young*	1
840	C31,C43,*Outside*,*Seroconverted*,*Young*	1
841	C31,C32,*Outside*	1
842	C19,C34,*Seroconverted*,*Young*	1
843	C2,C29,*Outside*,*Seroconverted*,*Young*	1
844	C32,*Outside*,*Seroconverted*	1
845	C17,C31,*Seroconverted*,*Young*	1
846	C13,C7,*Young*	1
847	C22,C36	1
848	C33,C6,*Outside*,*Young*	1
849	C35,C5,*Young*	1
850	C22,C34,*Seroconverted*,*Young*	1
851	C2,*Outside*,*Seroconverted*	1
852	C17,C32	1
853	C21,C6,*Seroconverted*,*Young*	1
854	C24,C9,*Outside*	1
855	C18,C32	1
856	C1,C16,C20,*Young*	1
857	C33,C42,*Outside*,*Young*	1
858	C39,C40,*Young*	1
859	C14,C3,*Young*	1
860	C21,C42	1
861	C2,C6,*Seroconverted*,*Young*	1
862	C33,C4,*Seroconverted*,*Young*	1
863	C27,C38,*Seroconverted*,*Young*	1
864	C15,C34,*Seroconverted*,*Young*	1
865	C23,C42,C6,*Outside*,*Seroconverted*,*Young*	1
866	C16,C2,C34,*Outside*,*Seroconverted*,*Young*	1
867	C44,C45,*Seroconverted*,*Young*	1
868	C12,C27,*Outside*	1
869	C21,C36	1
870	C27,C9,*Outside*,*Young*	1
871	C29,C9,*Young*	1
872	C12,C21,*Outside*,*Seroconverted*,*Young*	1
873	C40,C7,*Outside*,*Young*	1
874	C39,C40,*Outside*	1
875	C39,C40,*Outside*,*Seroconverted*,*Young*	1
876	C12,C29,*Seroconverted*,*Young*	1
877	C21,C40,*Outside*,*Seroconverted*,*Young*	1
878	C19,C35,*Outside*,*Young*	1
879	C16,C34,C7,*Outside*,*Young*	1
880	C10,C37,*Outside*,*Young*	1
881	C42,*Outside*,*Seroconverted*	1
882	C36,C8	1
883	C3,*Outside*	1
884	C22,C37,C7,*Outside*,*Young*	1
885	C18,C38,*Young*	1
886	C22,C7,*Outside*,*Young*	1
887	C36,C6,*Outside*,*Young*	1

Table F – continued from previous page

Id.	Variables with level "yes"	Count
888	C6,C7,*Outside*,*Seroconverted*,*Young*	1
889	C3,C4,*Young*	1
890	C20,C24,*Seroconverted*,*Young*	1
891	C34,C36,*Outside*,*Young*	1
892	C13,C5,*Seroconverted*,*Young*	1
893	C23,C6,*Young*	1
894	C15,C45,*Young*	1
895	C2,C31,C39,*Outside*,*Young*	1
896	C22,C32,*Outside*,*Young*	1
897	C14,C4,*Outside*,*Young*	1
898	C3,C34,*Outside*,*Young*	1
899	C2,C31,*Outside*,*Young*	1
900	C16,C6,*Outside*,*Seroconverted*,*Young*	1
901	C31,C34,*Seroconverted*,*Young*	1
902	C1,C3,*Young*	1
903	C1,C20,*Outside*,*Young*	1
904	C19,C33,*Young*	1
905	C16,C3,*Young*	1
906	C3,C5,*Outside*,*Young*	1
907	C42,C7,*Young*	1
908	C39,C44	1
909	C18,C7	1
910	C21,C30,*Outside*,*Young*	1
911	C12,C27,*Seroconverted*,*Young*	1
912	C3,C4	1
913	C28,C6,*Seroconverted*,*Young*	1
914	C1,C32	1
915	C34,C4,*Seroconverted*,*Young*	1
916	C24,C40,*Outside*,*Young*	1
917	C11,C19,*Outside*,*Seroconverted*,*Young*	1
918	C29,C4,*Young*	1
919	C18,C39	1
920	C20,C39,*Outside*,*Young*	1
921	C14,C21,*Outside*,*Young*	1
922	C19,C36	1
923	C19,C36,*Outside*,*Young*	1
924	C14,C7,*Outside*,*Seroconverted*	1
925	C19,C8,*Outside*,*Seroconverted*,*Young*	1
926	C20,C36,*Outside*,*Seroconverted*,*Young*	1
927	C17,C18,*Young*	1
928	C13,C45,*Outside*,*Seroconverted*,*Young*	1
929	C27,C5,*Seroconverted*,*Young*	1
930	C18,C32,*Young*	1
931	C14,C44,*Outside*,*Young*	1
932	C22,C36,*Outside*,*Seroconverted*,*Young*	1
933	C39,C4,*Seroconverted*,*Young*	1
934	C15,C30,*Seroconverted*,*Young*	1
935	C16,C28,C9,*Outside*,*Seroconverted*,*Young*	1
936	C16,C34,*Young*	1
937	C31,C7,*Outside*,*Young*	1
938	C19,C45,*Outside*,*Young*	1
939	C15,C4,*Outside*,*Seroconverted*,*Young*	1

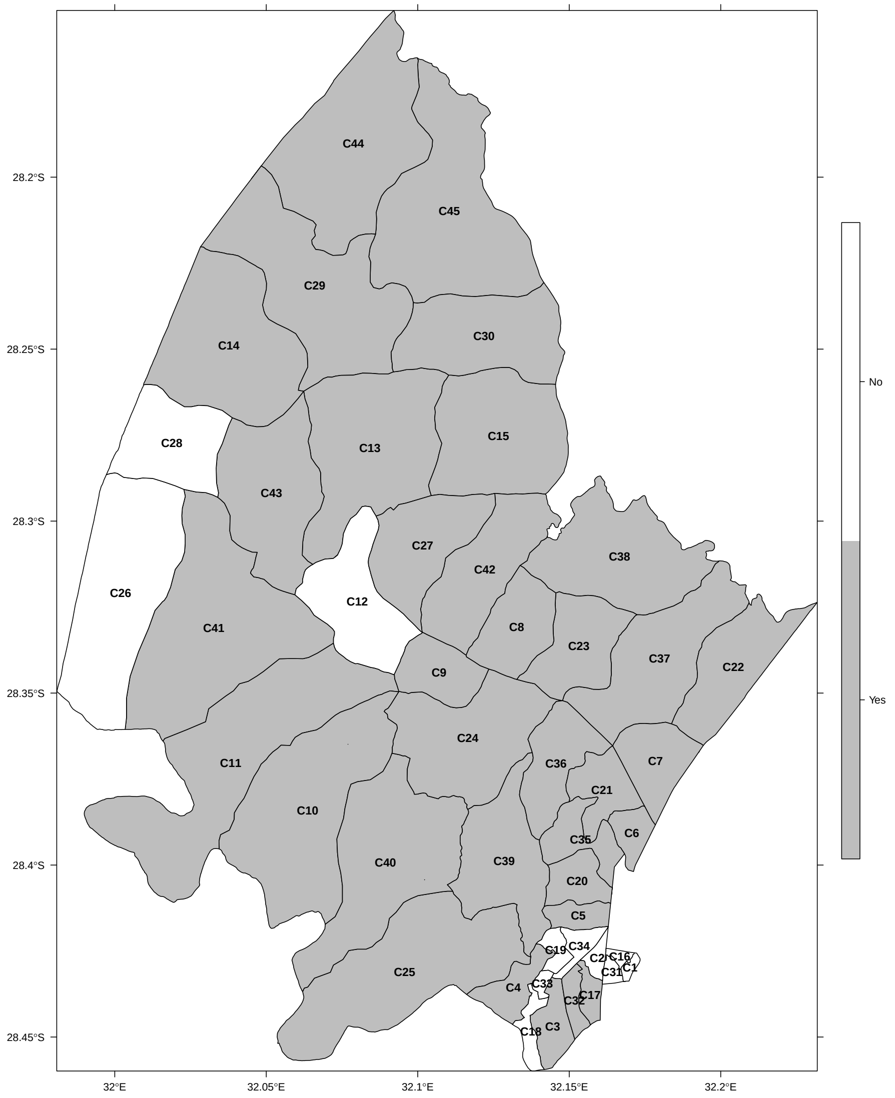

FIG. E. Map of the communities that are linked with an edge with the vertex *Outside* in the estimated conditional independence graph for men's mobility. A number of 33 communities (gray) are linked with *Outside*, while 12 communities (white) are not linked with an edge with *Outside*.

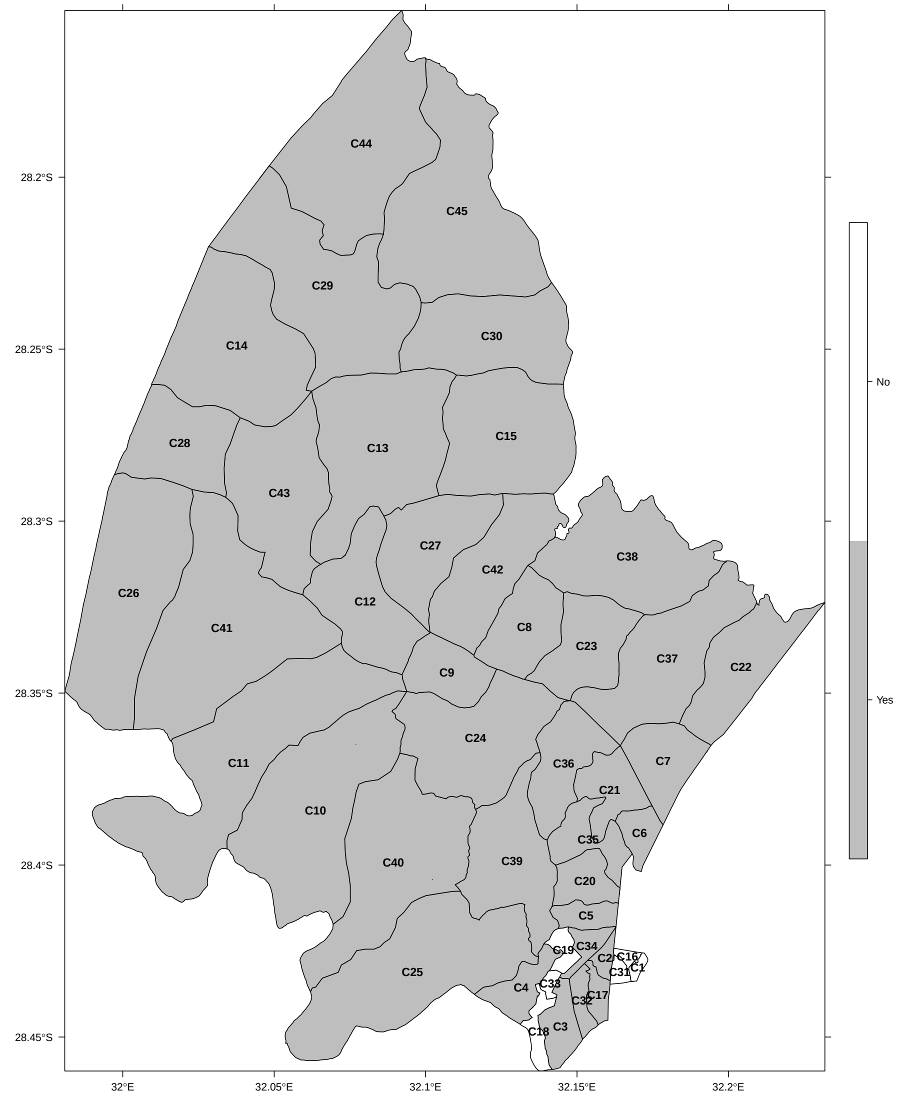

FIG. F. Map of the communities that are linked with an edge with the vertex *Outside* in the estimated conditional independence graph for women's mobility. A number of 39 communities (gray) are linked with *Outside*, while 6 communities (white) are not linked with an edge with *Outside*.

	Model	Dev	DF	p-value	AIC	BIC
1	*Seroconverted*<br>*Outside*<br>*Young*	89.35	4	<0.001	22095.90	22117.17
2	*Seroconverted – Outside*<br>*Young*	14.48	3	0.002	22023.03	22051.39
3	*Seroconverted*<br>*Outside – Young*	76.98	3	<0.001	22085.54	22113.89
4	*Seroconverted – Young*<br>*Outside*	88.17	3	<0.001	22096.72	22125.08
5	*Seroconverted – Outside*<br>*Young – Seroconverted*	13.30	2	0.001	22023.85	22059.30
6	*Seroconverted – Outside*<br>*Outside – Young*	2.11	2	0.348	–	–
7	*Outside – Young*<br>*Young – Seroconverted*	1.25	2	<0.001	22086.36	22121.80
8	*Seroconverted – Outside*<br>*Young – Seroconverted*<br>*Outside – Young*	1.53	1	0.215	–	–

TABLE C. The fit of three-way loglinear models for the cross-classification of 8,857 men from Table A. "Dev" stands for deviance, and "DF" stands for degrees of freedom. The loglinear models are specified by their maximal interaction terms.

	Model	Dev	DF	p-value	AIC	BIC
1	*Seroconverted*<br>*Outside*<br>Young	649.72	4	<0.001	38238.54	38260.75
2	*Seroconverted – Outside*<br>*Young*	649.43	3	<0.001	38195.24	38224.87
3	*Seroconverted*<br>*Outside – Young*	331.25	3	<0.001	37877.07	37906.69
4	*Seroconverted – Young*<br>*Outside*	381.61	3	<0.001	37927.43	37957.05
5	*Seroconverted – Outside*<br>*Young – Seroconverted*	336.32	2	<0.001	37884.14	37921.17
6	*Seroconverted – Outside*<br>*Outside – Young*	285.96	2	<0.001	37833.78	37870.81
7	*Outside* – Young<br>*Young – Seroconverted*	18.15	2	<0.001	37565.96	37602.99
8	*Seroconverted – Outside*<br>*Young – Seroconverted*<br>*Outside* – Young	1.25	1	0.264	–	–

TABLE D. The fit of three-way loglinear models for the cross-classification of 12,158 women from Table B. "Dev" stands for deviance, and "DF" stands for degrees of freedom. The loglinear models are specified by their maximal interaction terms.
